# Modeled sustainability impacts of increasing pork consumption among adults in the United States

**DOI:** 10.3389/fnut.2024.1508601

**Published:** 2025-01-17

**Authors:** Zach Conrad, Vincent Repoulis, Catherine Zavela

**Affiliations:** ^1^Department of Kinesiology, William & Mary, Williamsburg, VA, United States; ^2^Global Research Institute, William & Mary, Williamsburg, VA, United States

**Keywords:** pork, protein food, sustainability, food system, greenhouse gas

## Abstract

**Introduction:**

Little is known about the role of pork in sustainable diet patterns, given that it is often aggregated with other animal proteins or not evaluated at all. To address this gap, this study modeled the sustainability impacts of replacing different protein foods with pork in a nationally representative sample of adults in the United States (US).

**Methods:**

Data on dietary intake, greenhouse gas emissions (GHGE), cumulative energy demand, water scarcity footprint, land, pesticides, fertilizer nutrients, food prices, and diet quality were obtained from publicly available repositories. A food substitution model was constructed to evaluate the change in each sustainability impact when 1–3 servings of beef, poultry, seafood, eggs, or legumes were replaced by pork.

**Results:**

Modeled substitution of beef with pork was associated with reductions in GHGE, land, pesticides, and fertilizer nutrients by 11–35%, and substitution of seafood with pork was associated with reductions in cumulative energy demand by 6% and diet cost by <1%. All other substitutions led to an increase in sustainability impacts of up to 5%, including all outcomes associated with substituting poultry, eggs, and legumes with pork.

**Discussion:**

The US federal government can play an important role in improving data collection methods that distinguish between pork and other meats. This can facilitate further research to evaluate sustainability trade-offs, which can inform clinical practice and public policy to support informed food choices for consumers.

## 1 Introduction

Current food systems present challenges for all domains of sustainability, which include nutrition/health, environment, economy, and society. In the United States (US), less than 5% of the population meets dietary recommendations (1), which accounts for nearly half of all deaths from cardiometabolic diseases (2) and is the leading modifiable risk factor for overall mortality (3). Suboptimal diet quality also accounts for 11% of disability-adjusted life years (3) and nearly 20% of total direct medical costs (4). Healthier diets are often more expensive than less healthy ones (5) and are unaffordable for lower income minority groups (6), which has contributed to worsening disparities in diet quality (1) and healthcare costs (4). At the same time, the US food system places a heavy burden on the environment. It is responsible for nearly 20% of total national greenhouse gas emissions (GHGE) (7) and over 25% of land use and freshwater withdrawals (7). On a per capita basis, GHGE are over 70% higher in the US than the global average (8).

There is growing urgency among consumers (9), clinicians (10), policymakers (11), and other stakeholders (12) to identify sustainable food choices to stay within planetary boundaries. There is a heightened focus on dietary shifts within the protein foods group (12), given the wide range of sustainability impacts across different foods within this category (13). Prior modeling research has focused on replacing beef with poultry or plant proteins, or replacing animal proteins with plant proteins, which have resulted in higher diet quality and reductions in GHGE and diet cost (14–16).

However, less is known about the role of pork in sustainable diet patterns, given that it is often aggregated with other animal proteins or not evaluated at all (17). This oversight prevents a comprehensive understanding of how dietary shifts within the protein food category can impact sustainability outcomes, given that pork has a prominent place in US diets. Pork accounts for nearly 25% of daily intake of meat and poultry by weight (1.45 oz), and consumption has increased by 14% since 2014 (18). Pork also has a central place in consumer diets throughout the world, which is supported by US production and exports. Pork is the most consumed meat in the world, exceeding 115 million tons annually (19), and consumption is expected to grow by 8% by 2033 (20). The US is the third largest producer of pork, accounting for 11% of global production (21), and the second largest exporter of pork, accounting for 31% of exports (19).

Prior research has shown that pork consumption is associated with greater likelihood of meeting many micronutrient requirements (22), and has a higher digestible indispensable amino acid score (DIAAS) than most other protein foods (23). Pork also outperforms some other protein foods in other sustainability metrics (13, 17). For example, GHGE from pork (per kg of food) are 6-fold lower than for beef (13), and pork is the lowest cost protein food (per 50 g of protein) behind eggs and beans (17). Thus, further research is needed to evaluate whether replacing different protein foods with pork can improve sustainability outcomes.

To address this gap, the present study models the sustainability impacts of replacing beef, poultry, seafood, eggs, and legumes with pork in a nationally representative sample of US adults. Sustainability is comprehensively evaluated using multiple impacts to understand trade-offs, which include GHGE, cumulative energy demand, water scarcity footprint, agricultural land, fertilizer nutrients, pesticides, diet cost, and diet quality. This research can help inform important clinical and policy discussions related to diet sustainability in the US (11, 24).

## 2 Methods

### 2.1 Dietary data

Data on individual-level intake of foods and nutrients, as well as sociodemographic characteristics, were acquired from the National Health and Nutrition Examination Survey (NHANES), 2011–2018 (25). Data are collected continuously from approximately 5,000 non-institutionalized participants each year using a clustered, stratified, multi-stage sampling design, and data are released in two-year cycles (25). Some demographic groups are oversampled to increase reliability and precision for subgroup analysis (26). Dietary data collection is administered by a trained interviewer that uses the Automated Multiple Pass Method, a computer-assisted 24-h recall, to minimize participant burden and increase reliability and validity of the data (27, 28). Data from one day of dietary recall was appropriately used to estimate per capita intake (29).

### 2.2 Food categories and serving sizes

As part of the automated dietary recall procedure, each food (including mixed dishes) reported consumed by NHANES participants is automatically assigned an 8-digit numerical identifier (known as a *food code*) based on the predominant ingredient in that food, using the US Department of Agriculture (USDA) Food and Nutrient Database for Dietary Studies (FNDDS) (30). In the present study, the FNDDS food codes from 2011–2018 were used to group foods into the following protein dish categories: beef, pork, poultry, seafood, eggs, and legumes (Supplementary Table S1). Foods that are typically consumed in small amounts were excluded from these categories because their serving sizes are not comparable to other foods within these categories, such as bacon (beef, pork, and Canadian), dried beef, spareribs, cracklings, pork skin, and miscellaneous parts. Foods that contain multiple types of meat were also not assigned to a food category. However, this study included the sustainability impacts of all foods when estimating total daily per capita impacts. For each protein dish category, the median gram weight across all eating occasions for all foods was used to represent the median serving size (Supplementary Table S2).

### 2.3 Diet quality measurement

The Healthy Eating Index-2020 (HEI-2020) was used to measure diet quality because it measures adherence to the Dietary Guidelines for Americans (31). The HEI-2020 includes nine components to encourage (total fruit, whole fruit, total vegetables, greens and beans, whole grains, dairy, total protein foods, seafood and plant proteins, and the ratio of unsaturated to saturated fats) and four components to limit (refined grains, sodium, added sugars, and saturated fats). The intake of most components is energy-adjusted to 1,000 kcal, and is scored against predefined minimum and maximum values, with intermediate intakes scored proportionally (Supplementary Table S3). Each component is scored from 0–5 or 0–10, and higher scores represent more favorable intakes. For each participant, scores for all components are summed to generate a total score out of 100 (31).

### 2.4 Greenhouse gas emissions, cumulative energy demand, and water scarcity footprint

The database of Food Impacts on the Environment for Linking to Diets (dataFIELD) provided information on GHGE, cumulative energy demand (CED), and water scarcity footprint (WSF) for each food in NHANES (13, 32). These data were compiled from 321 food environmental life cycle assessments (LCA) published from 2005–2016 using a systematic review, which resulted in 1,645 combinations of food types and production scenarios (33). Data represent most regions of the world, with the majority from Europe (33). Nearly all studies accounted for agricultural production, 51% accounted for post-farmgate processing, 19% accounted for distribution and retail, and 6% accounted for the consumer-level impacts. For each food, environmental impacts from multiple studies were averaged and matched to commodities in the US Environmental Protection Agency (EPA) Food Commodity Intake Database (FCID), which provides information on the amount of approximately 500 commodity ingredients in each food in NHANES (34). The present study used an updated version of FCID that aligns with NHANES 2011–2018 (35).

### 2.5 Agricultural land, fertilizer nutrients, and pesticides

Foodprint 2.0 was used to estimate the agricultural resource requirements associated with individual-level diet patterns in NHANES (36). These agricultural resources include land (including all types of cropland and pasture land), fertilizer nutrients (sum of nitrogen, phosphorus-P_2_O_5_, potash-K_2_O, and sulfur), and pesticides (sum of herbicides, insecticides, and fungicides). Foodprint 2.0 is a biophysical simulation model that represents the US food system as a series of integrated processes. Embedded data and calculations are used to transform NHANES foods in their as-consumed forms into agricultural commodities and the agricultural resources needed to produce these commodities (Supplementary material 1). Foodprint 2.0 accounts for population size, international food trade, loss and waste, food composition, food processing conversions, livestock feed requirements, crop and livestock yields, availability of agricultural land, suitability of agricultural land for food production, multi-use crops (i.e., crops that are used to produce multiple products from equivalent mass), multi-use cropland (i.e., cropland used to produce multiple crops during different parts of the year), and application rates for fertilizer nutrients and pesticides. All parameters represent US national averages.

### 2.6 Diet cost

The USDA Economic Research Service (ERS) Purchase-to-Plate Price Tool (PPPT) provided information on prices for each NHANES food (37). These data were collected from retail checkout scanners and represent nearly 50% of all retail food sales in the US (38). USDA ERS staff matched these scanner data to NHANES foods using machine learning, and removed the cost associated with loss and waste so the final data reflect the cost associated with the consumed portion only (39).

Food prices from PPPT only represent food-at-home (FAH) prices and there are no publicly available data on national average food-away-from-home (FAFH) prices for each NHANES food. Therefore, PPPT assigns FAH prices for all NHANES foods regardless of whether participants reported consuming that food at home or away from home. This will severely underestimate total diet cost because consumers typically face higher prices for FAFH than FAH, and other data show that FAFH accounts for approximately 50% of consumer food expenditures (40). Therefore, the present study derived FAFH prices using a methodology previously demonstrated (5, 41) and described below.

Data on FAH and FAFH spending were acquired from the National Household Food Acquisition and Purchase Survey (FoodAPS) (42), and were used to derive a coefficient that converted FAH prices (from PPPT) to FAFH prices for each of the FAFH reported consumed by NHANES participants. From April 2012 through January 2013, FoodAPS used a multi-stage survey design to collect information from US households on the price of all purchased foods from receipts and scanned barcodes (42). Survey-weighted mean FAH and FAFH prices were estimated for each major food group (meat, poultry, seafood, eggs, dairy, fats and oils, fruits and vegetables, sweets, grains, non-alcoholic beverages, and other foods), and these were used to derive a coefficient that represents the ratio of FAFH-to-FAH prices for each food group. These coefficients were multiplied by the price of each FAFH in PPPT to estimate its FAFH price. For example, if the price of a given beef product was $3.50 (from PPPT), and if the mean price of FAFH beef was 3.17 times greater than the mean price of FAH beef (from FoodAPS), the adjusted price of that given beef product would be estimated as $11.11 ($3.50 × 3.17).

### 2.7 Impacts per gram and per serving

The price, GHGE, CED, WSF, land, fertilizer nutrients, and pesticides per gram of each NHANES food were calculated by dividing the total impacts associated with each food by its gram weight. The amount of each HEI-2020 component per gram of each NHANES food was also calculated in the same way. After accounting for food loss and waste (see below), these were multiplied by the median serving size (in gram weight) of each protein dish category to estimate the impacts per serving of each category (Supplementary Tables S4, S5).

### 2.8 Food loss and waste

Data on GHGE, CED, and WSF (from dataFIELD); and food prices (from PPPT); do not include the environmental impacts and cost associated with food that is lost and wasted at the retail and consumer levels. Not accounting for food loss and waste can underestimate consumer food demand (43), environmental impacts (44), and diet cost (41) by up to 37–42%. To fill these gaps, data on commodity-level loss and waste were acquired from the USDA Loss-adjusted Food Availability data system (LAFA) (45), and matched to commodities in the FCID, which links to NHANES. This procedure has been demonstrated previously (5, 41, 43, 44, 46, 47) and additional details on sources of uncertainty and embedded assumptions are described elsewhere (5, 47).

### 2.9 Diet modeling

A food substitution model was developed to estimate the effects of iteratively replacing 1–3 servings of each protein dish (beef, poultry, seafood, eggs, and legumes) with 1–3 servings of a pork dish on daily per capita sustainability impacts, which include GHGE, CED, WSF, land, fertilizer nutrients, pesticides, diet cost, and diet quality. Substitutions were performed separately for each protein dish. The serving size of each protein dish varied from 92 g (eggs) to 117 g (beef), so the serving size used for pork varied accordingly (Supplementary Table S2). For example, each serving of beef (117 g) was replaced by 117 g of pork, whereas each serving of poultry (110 g) was replaced by 110 g of pork. These substitutions were made based on observed serving sizes (i.e., from NHANES 24-h recalls) to reflect the observed quantity of dishes consumed during eating occasions in free living conditions, which increases generalizability. Substitutions were only made for whole servings. For example, participants who consumed 1.1 servings of beef at baseline experienced a one-serving substitution for pork, and participants who consumed 3.1 servings of beef experienced a three-serving substitution for pork. This modeling structure allows for discretionary intake of foods, which may be more practical in real-world settings than their complete elimination.

### 2.10 Sensitivity analyses

To evaluate the robustness of the modeling procedure, sensitivity analyses were used to understand whether recommended serving sizes (rather than observed serving sizes) produced different results. Each dish consumed by NHANES participants was assigned a serving size based on the Reference Amounts Customarily Consumed (RACC) established by the US Food and Drug Administration (FDA; Supplementary Table S2) (48).

### 2.11 Statistical analyses

Mean daily per capita GHGE, CED, WSF, land, fertilizer nutrients, pesticides, diet cost, and diet quality were estimated at baseline and after modeled substitutions. Means were estimated using linear regression models adjusted for energy intake (continuous) and NHANES survey cycle (continuous). Differences in mean impacts between baseline and modeled substitutions were tested using paired Wald tests at *p* < 0.0125 (Bonferroni correction: 0.05 ÷ 3 pairwise tests of same comparator): baseline vs. 1-serving substitution, 1-serving substitution vs. 2-serving substitution, and 2-serving substitution vs. 3-serving substitution. NHANES design variables and survey weights were used to account for the complex sampling design and to produce nationally representative estimates. Stata 16.1 (49) was used for data management and analyses.

## 3 Results

### 3.1 Participant characteristics

A total of 33,325 NHANES participants provided dietary data from 2011–2018. Participants were excluded from the analysis if they were < 20 y (*n* = 13,719), did not consume any dietary energy (kcal) (*n* = 1), or had ≥1 sustainability impact (GHGE, CED, WSF, land, fertilizer nutrients, pesticides, or diet cost) that was >3SD from the mean (*n* = 2,122). The final analytic sample included 17,483 participants. The mean age of participants was 48 y (Table 1), and more than half (55%) were female. The majority had an income-to-poverty ratio ≥ 1.86 (61%) and were Non-Hispanic White (65%).

**Table 1:** Characteristics of study participants, 2011–2018 (*n* = 17,483).

Characteristic	% (95% CI)^1^
Age, y (mean)	48.3 (47.6–49)
Gender	
Male	45.4 (44.4–46.4)
Female	54.6 (53.6–55.6)
Income-to-poverty ratio	
≤1.30	21.3 (19.7–23.1)
1.31–1.85	10.1 (9.3–11)
1.86–4.99	37.3 (35.4–39.2)
≥5.00	23.9 (21.8–26)
Missing	7.4 (6.6–8.3)
Race and Hispanic origin	
Non-Hispanic White	65.0 (61.4–68.4)
Non-Hispanic Black	11.5 (9.7–13.6)
Hispanic^2^	14.4 (12.2-16.9)
Non-Hispanic Asian	5.7 (4.7–6.8)
Other^3^	3.4 (3-3.9)

Sample sizes are unweighted. ^1^Unless otherwise noted. ^2^Includes Mexican American. ^3^Includes multi racial.

### 3.2 Environmental impacts: greenhouse gas emissions, cumulative energy demand, and water scarcity footprint

**Figure 1:**
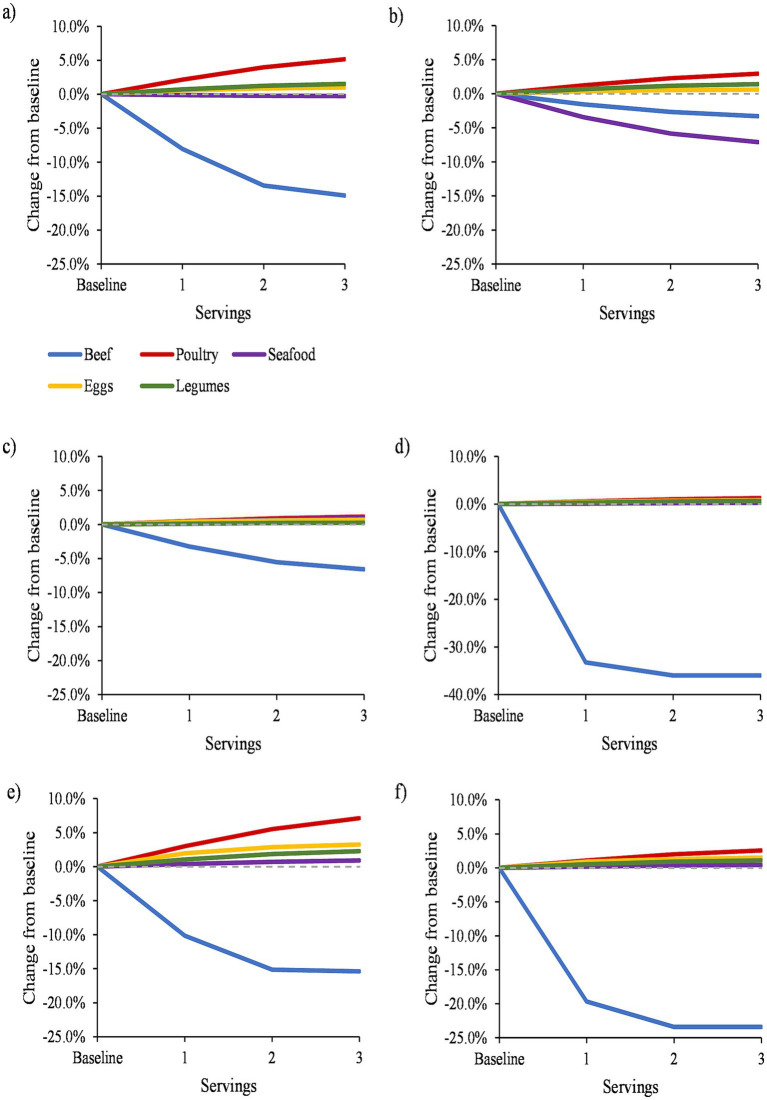
Percent change in mean daily **(A)** greenhouse gas emissions, **(B)** cumulative energy demand, **(C)** water scarcity footprint, **(D)** land, **(E)** fertilizer nutrients, and **(F)** pesticides at baseline and after modeled replacement of protein foods with pork, 2011–2018 (*n* = 17,483). All results were adjusted for energy intake (kcal) and survey cycle using linear regression models. Dashed horizontal line represents no difference. Solid lines that appear above the dashed line indicate that replacing a given protein food with pork led to an increase in environmental impacts, and solid lines that appear below the dashed line indicate that replacing a given protein food with pork led to a decrease in environmental impacts.

The mean daily intake (in servings) of beef, pork, poultry, seafood, eggs, and legume dishes at baseline and after modeled substitutions are presented in Supplementary Table S6. GHGE decreased when replacing up to 3 servings of beef (15% decrease) and seafood (<1% decrease) with pork (Figure 1A; Supplementary Table S7; *p* < 0.001 for all comparisons). Replacing up to 3 servings of poultry, seafood, eggs, and legumes with pork led to an increase in GHGE of up to 5%, with the greatest increase observed for poultry substitution (*p* < 0.001 for all comparisons). CED decreased when replacing up to 3 servings of beef (3% decrease) and seafood (7% decrease), and increased by up to 3% when replacing poultry, eggs, and legumes (Figure 1B; *p* < 0.001 for all comparisons). WSF decreased by up to 6% when replacing beef and increased by up to 1% when replacing each of the other protein foods (Figure 1C; *p* < 0.001 for all comparisons).

### 3.3 Agricultural resource use: land, fertilizer nutrients, and pesticides

Replacing up to 3 servings of beef with pork led to a decrease in land use, fertilizer nutrients, and pesticides by up to 35%, 11%, and 17%, respectively (Figures 1D–F; Supplementary Table S7; *p* < 0.001 for all comparisons). Substituting poultry, seafood, eggs, or legumes was associated with a ± 1% change in land use, fertilizer nutrients, and pesticides (p < 0.001 for all comparisons).

### 3.4 Diet cost and diet quality

**Figure 2:**
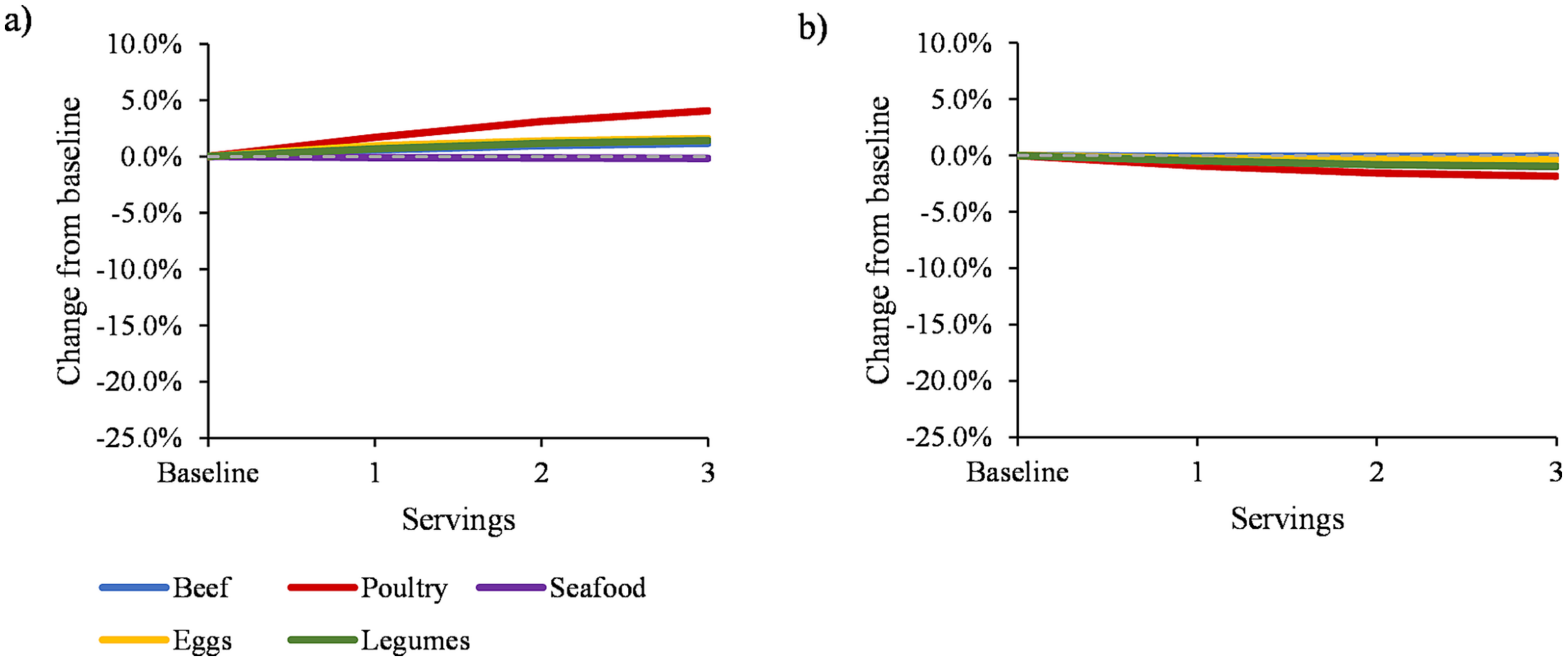
Percent change in mean daily **(A)** diet cost, and **(B)** Healthy Eating Index-2020 score at baseline and after modeled replacement of protein foods with pork, 2011–2018 (*n* = 17,483). All results were adjusted for energy intake (kcal) and survey cycle using linear regression models. Dashed horizontal line represents no difference. Solid lines that appear above the dashed line indicate that replacing a given protein food with pork led to an increase in diet cost or diet quality, and solid lines that appear below the dashed line indicate that replacing a given protein food with pork led to a decrease in diet cost or diet quality.

Diet cost decreased by <1% when replacing beef or seafood with pork, and increased by up to 5% when replacing poultry, eggs, or legumes (Figure 2A; Supplementary Table S7). HEI-2020 scores decreased by up to 2% after replacing poultry with pork, and decreased by <1% after replacing beef, seafood, eggs, or legumes (Figure 2B). Each protein dish category represents a composite of all foods within that category, including foods with multiple ingredients. Thus, protein dish substitutions can lead to changes in intake of non-protein foods. Supplementary Table S8 and Supplementary Figure S1 show how these foods influence diet quality by evaluating the impact of protein dish substitutions on HEI-2020 component scores. Substituting 1–3 servings of each protein dish for pork was associated with lower intake of refined grains (up to 4% higher HEI-2020 component score), and greater intake of total protein (up to 2% higher score). For most protein dish substitutions, sodium intake increased (up to 9% higher score), saturated fat intake increased (up to 7% higher score, except lower scores for beef and eggs), unsaturated fat intake decreased (up to 7% lower score, except higher score for beef), and consumption of seafood and plant proteins decreased (up to 6% lower score). Smaller decreases in intake were observed for total vegetables and dairy (up to 2% lower score for each) after replacing each protein dish with pork.

### 3.5 Sensitivity analyses

In sensitivity analyses, serving sizes were estimated using the RACCs published by the US FDA, which represent recommended serving sizes rather than observed serving sizes (48). In several cases, RACC serving sizes produced changes in magnitude compared to the main results (Supplementary Table S9). For beef substitutions, GHGE, land, and pesticides were up to 8% lower than the main results. For poultry, seafood, and egg substitutions, land, fertilizer nutrients, and pesticides were up to 9% higher than the main results. For all other cases, the change was ±1% compared to the main results.

## 4 Discussion

In this nationally representative study of over 17,000 US adults, modeled substitution of protein foods (beef, poultry, seafood, eggs, and legumes) with pork led to heterogeneous changes in sustainability impacts. The greatest changes were observed when beef was replaced with pork, which was associated with reductions in GHGE, land, pesticides, and fertilizer nutrients by 11–35%. Substituting seafood with pork led to reductions in CED by 7% and diet cost by <1%. All other substitutions led to an increase in sustainability impacts of up to 5%. These findings can help inform clinical, policy, and research discussions about sustainable diet patterns, which have previously not focused on the role of pork as a distinct protein food.

Despite the prominence of pork in consumer diets, it is often not disaggregated from beef in research and policy documents (17). This has led to gaps in knowledge about the role of pork in sustainable diet patterns, which limits policy action. Although some prominent food frequency questionnaires do distinguish between pork and beef (50), others do not (51). Pork and beef are grouped into the categories *meat* and *cured meat* in the Food Patterns Equivalents Database (FPED), which is relied upon by researchers to monitor the dietary intake of the US population (52). The implications are widespread, given that these findings are featured in the Dietary Guidelines for Americans, which informs all federal policies and programs (53). For example, pork and beef are aggregated into a single category in the USDA Thrifty Food Plan, which is used to calculate monthly benefits for over 40 million people that participate in the Supplemental Food Assistance Program (54).

The US federal government can play an important role in improving data collection methods that distinguish between pork and other meats. The National Strategy on Hunger, Nutrition, and Health (hereafter, the *National Strategy*), published by the White House in 2022, provides the justification for this effort (11). The National Strategy calls for a whole-of-society approach to improve the performance of the US food system on all domains of sustainability. It is divided into five thematic “pillars,” one of which is “Pillar 5-Enhance Nutrition and Food Security Research,” which recommends improvements to data metrics, data collection, and research that can be used to inform nutrition and food security policy (11). For example, it specifically calls for a new iteration of the National Household Food Acquisition and Purchase Survey (FoodAPS), last updated in 2013 (42), which provides novel information on community food environments and food spending. FoodAPS has been a critical data source for estimating diet costs (46), including for the present study. The National Strategy can also provide the impetus for refining FPED so that it disaggregates pork from other meats, and can be used to justify a much-needed update of the Food Intakes Converted to Retail Commodities Database (FICRCD) and the Food Commodity Intake Database (FCID) (55). The FICRCD (last updated in 2013) (56) and FCID (last updated in 2010) (34) are commodity ingredient databases that are used to disaggregate the ingredients in each NHANES mixed dish so they can be linked with sustainability impacts, and both of these disaggregate pork from other meats. Regular updates to these essential databases are needed to fill knowledge gaps about sustainable food substitutions, especially in the protein foods category.

The present study also has implications for clinical practice. We found that substituting pork for other protein foods was associated with lower intake of refined grains and modestly higher intake of protein, but increased intake of sodium. These findings are consistent with prior research that showed that pork consumers (all, fresh, and processed pork) were more likely to exceed the daily recommended intake for sodium (22). These findings demonstrate that pork is a carrier food that presents trade-offs for diet quality, and highlights the value of examining consumption of mixed dishes rather than individual foods, which reflects real-world eating conditions. Clinicians can play an important role in counseling their patients to modify their pork dishes to improve overall diet quality, which should focus on reducing sodium, or by replacing pork dishes with lower-sodium protein options. Pork is a common ingredient in processed meats, such as sausage, which typically contain high amounts of sodium, and are associated with elevated risk of cardiometabolic diseases and some types of cancers (57). Shifting to fresh pork, or to another lower-sodium protein option, may provide an opportunity to reduce sodium intake and improve overall diet quality.

At the same time, compared to non-pork consumers, those who consume pork (all types) are more likely to meet daily micronutrient recommendations for copper, iron, phosphorus, selenium, zinc, and most B vitamins (22). Compared to most other protein foods, pork is associated with greater digestible indispensable amino acid scores (DIAAS) (23) and greater postprandial bioavailability of essential amino acids in children and adults (58). Increasing pork consumption by one oz-equivalent per day was associated with up to 8% lower likelihood of any functional limitations among older adults (59). Pork is also the most affordable source of protein when measured as US$ per gram of protein, behind only beans and eggs (17), thus making it an economically accessible source of high quality nutrients.

Several recent studies have evaluated the health, environmental, and economic impacts of protein food substitutions, but these did not evaluate pork as a distinct food category (14–16). The present study fills this gap by showing that substituting different protein foods with pork can lead to heterogeneous changes in sustainability impacts. Reductions in sustainability impacts were only observed when beef was replaced by pork (11–35% lower GHGE, land, pesticides, and fertilizer nutrients) and when seafood was replaced by pork (7% lower CED and <1% lower diet cost). All other substitutions were associated with greater sustainability impacts of up to 5%, including all outcomes associated with substituting poultry, eggs, and legumes with pork.

Some of these findings differ from prior modeling research that showed that diet costs can theoretically be lowered while meeting energy and nutrient requirements if consumers chose the lowest price pork option rather than beef (60). This approach differs from the present study, which evaluated the price of pork products actually purchased by consumers, rather than the lowest cost option. Nevertheless, diet sustainability trade-offs are frequently observed in the research literature. For example, a recent review showed that a shift toward more environmentally friendly diets may lower the intake of some micronutrients (61). The Eat-Lancet diet, which was designed to represent healthy and sustainable diet patterns (62), has been associated with higher land use (63) and lower intake of some micronutrients (64), and is unaffordable for people living in lower income regions of the world (65). In the US, other research showed that higher diet quality was associated with higher amounts of food waste (47), food spending (5, 46), and some types of agricultural resources (43), and diets with lower GHGE have been associated with lower intake of some micronutrients (66). These trade-offs raise important questions for researchers, clinicians, policymakers, and other stakeholders about how to communicate this nuanced information to the public, and how consumers can use this information to make sustainable food choices in different contexts.

This study has several strengths. The dietary data were nationally representative, making these findings generalizable to the US adult population. Food substitutions were made on the basis of observed serving sizes, rather than recommended serving sizes, which reflects the real-world conditions in which individuals make food choices. Sensitivity analyses were used to test the robustness of this approach by using recommended serving sizes established by the US FDA, which revealed several differences in the results, although most of these were modest in scale (±5%). Finally, this study evaluated eight sustainability impacts that represent three of the four domains of sustainability (nutrition/health, environment, and economic), which allowed us to examine sustainability trade-offs.

This study also has several limitations. NHANES participants typically report consuming mixed dishes that contain multiple ingredients, so each dish was categorized according to its primary food component, and the sustainability impacts were averaged across all dishes within each category. Therefore, it is possible that the results were influenced by secondary ingredients in each dish (i.e., those that represented a smaller share of each dish on a weight basis). However, this reflects many real-world conditions in which consumers replace one type of dish with another type of dish, rather than substituting individual ingredients. For example, it is unlikely that most consumers would use pork rather than eggs in an omelet, although they may use pork rather than beef. There is also wide variation in sustainability impacts by cuts of pork (67), geographic conditions, and other factors (68) that could not be measured but may be an important avenue for future research. Finally, due to lack of data availability, some sustainability impacts could not be measured, including some environmental impacts (69), antimicrobial use (70), ethical treatment of animals (71), and forced labor (72).

## 5 Conclusion

Modeled substitution of different protein foods with pork led to heterogeneous changes in sustainability impacts. Reductions in sustainability impacts were observed when pork replaced beef (lower GHGE, land, pesticides, and fertilizer nutrients) and seafood (lower CED and diet cost). All sustainability impacts increased when pork replaced poultry, eggs, and legumes. This fills an important research gap because prior studies have typically not evaluated pork as a distinct protein food, and some major federal food policies do not distinguish pork from other meats. This limits consumers’ ability to make informed, sustainable food choices. The US federal government can play an important role in improving data collection methods that distinguish pork from other meats.

## Data Availability

The original contributions presented in the study are included in the article/Supplementary material, further inquiries can be directed to the corresponding author.
