# Corrigendum: Modeled sustainability impacts of increasing pork consumption among adults in the United States

**DOI:** 10.3389/fnut.2025.1623296

**Published:** 2025-05-28

**Authors:** Zach Conrad, Vincent Repoulis, Catherine Zavela

**Affiliations:** ^1^Department of Kinesiology, William & Mary, Williamsburg, VA, United States; ^2^Global Research Institute, William & Mary, Williamsburg, VA, United States

**Keywords:** pork, protein food, sustainability, food system, greenhouse gas

In the published article, there was an error in **Methods**, *Statistical analyses*, paragraph 1. The incorrect sentence read as “Differences in mean impacts between baseline and modeled substitutions were tested using paired Wald tests at P<0.025 (Bonferroni correction: 0.05 ÷ 2 pairwise tests of same comparator): baseline vs. 1-serving substitution, 1-serving substitution vs. 2-serving substitution, and 2-serving substitution vs. 3-serving substitution.” This should have been written as “Differences in mean impacts between baseline and modeled substitutions were tested using paired Wald tests at *P* < 0.0125 (Bonferroni correction: 0.05 ÷ 3 pairwise tests of same comparator): baseline vs. 1-serving substitution, 1-serving substitution vs. 2-serving substitution, and 2-serving substitution vs. 3-serving substitution.”

The authors apologize for this error and state that this does not change the scientific conclusions of the article in any way. The original article has been updated.

