# Supplementary material for: Modeled sustainability impacts of increasing pork consumption among adults in the United States
Source: Front Nutr. 2025 Jan 17;11:1508601. doi: 10.3389/fnut.2024.1508601 (PMC11783847; doi:10.3389/fnut.2024.1508601)
Supplement: Supplementary file 1 [file Data_Sheet_1.PDF]

Supplemental Table 1: Food categorization

Food and beverage category[1]	FNDDS code[2]
Beef	21, 25210210, 25210620, 25220105, 25220106, 25220108, 25220150, 25220390, 25220430, 25220470, 25221530, 25231110, 25231120, 25231150, 27460750, 27461010, 2711, 2721, 2731, 2741, 2751, 2756406, 2756407, 2756408, 2756409, 2756410, 2811
Pork	22, 25221405, 25221406, 25221408, 25221450, 25230210, 25230220, 25230230, 25230235, 25230420, 25230430, 25230530, 25240220, 2712, 2722, 2732, 2742, 2752
Poultry	24, 25210310, 25210410, 25220440, 25221830, 25221855, 25221860, 25230310, 25230320, 25230340, 25230780, 25230785, 25230790, 25230800, 25230820, 25230840, 25230900, 25230905, 25240110, 2714, 2724, 2734, 2744, 2754, 2756424, 2756425, 2756426, 2756427, 2756428, 2814
Seafood	26, 2715, 2725, 2735, 2745, 2755, 2815
Eggs	31, 321, 322, 324, 35
Legumes[3]	411, 412, 413

[1]Predominant ingredient in mixed dish.

[2]Leading digits in each 8-digit food code from the USDA Food and Nutrient Database for Dietary Studies (FNDDS).

[3]Includes Beans, peas, lentils, soybeans, and tofu. Excludes plant-based meat alternatives made from legumes (e.g., burgers).

Supplemental Table 2: Serving sizes of target and alternate foods, 2011-2018 (n=17,483)

Food category[1]	n[1]	Median observed serving size, grams (5th-95th percentile)	Mean RACC serving size, grams (5th-95th percentile)
Beef	5,489	117 (21-333)	90 (85-140)
Pork	5,438	N/A	89 (85-140)
Poultry	9,452	110 (26-327)	91 (85-140)
Seafood	3,204	99 (12-340)	89 (85-140)
Eggs	4,650	92 (33-226)	82 (50-110)
Legumes	2,406	98 (16-314)	96 (90-130)

[1]Number of eating occassions.

RACC, Recommended Amount Customarily Consumed. From the US Food and Drug Administration at: https://www.fda.gov/regulatory-information/search-fda-guidance-documents/guidance-industry-reference-amounts-customarily-consumed-list-products-each-product-category

Supplemental Table 3: Scoring standards for each component of the Healthy Eating Index-2020 (HEI-2020)

Component	Minimum score	Maximum score
Total fruit	No fruit (0 points)	≥0.8 cup equivalents (5 points)
Whole fruit	No whole fruit (0 points)	≥0.4 cup equivalents (5 points)
Total vegetables	No vegetables (0 points)	≥1.1 cup equivalents (5 points)
Dark green vegetables and legumes	No greens and beans (0 points)	≥0.2 cup equivalents (5 points)
Whole grains	No whole grains (0 points)	≥1.5 ounce equivalents (10 points)
Refined grains	≥4.3 ounce equivalents (0 points)	≤1.8 ounce equivalents (10 points)
Dairy	No dairy (0 points)	≥1.3 cup equivalents (10 points)
Total protein foods	No protein foods (0 points)	≥2.5 ounce equivalents (5 points)
Seafood and plant proteins	No seafood or plant proteins (0 points)	≥0.8 ounce equivalents (5 points)
Unsaturated fats	(PUFA + MUFA)/SFA≤1.2 (0 points)	(PUFA + MUFA)/SFA≥2.5 (10 points)
Saturated fats	≥16% of energy (0 points)	≤8% of energy (10 points)
Added sugars	≥26% of energy (0 points)	≤6.5 % of energy (10 points)
Sodium	≥2.0 grams (0 points)	≤1.1 grams (10 points)

EPA, eicosapentaenoic acid (20:5, n-3).

DHA, docosahexaenoic acid (22:6, n-3).

Development and validation available at https://epi.grants.cancer.gov/hei/developing.html#2015

Energy-adjusted to 1,000 kcal using the density method, except for unsaturated fatty acids, saturated fatty acids, and added sugars.

Supplemental Table 4: Mean environmental impacts and agricultural resource demand per serving of food, 2011-2018 (n=17,483)

Food category (serving size)[1]	GHGE (kg CO_2 eq)	CED (MJ)	WSF (L eq × 10^2)	Land (m^2)	Fertilizer nutrients (kg × 10^{-3})	Pesticides (kg × 10^{-3})	Price (US $)
				Mean per serving			
Beef (117 g)	3.37	7.30	10.28	39.54	40.77	1.54	4.48
Poultry (110 g)	0.46	3.20	2.47	0.65	11.28	0.16	2.79
Seafood (99 g)	0.85	15.75	0.95	0.44	6.54	0.11	3.83
Eggs (92 g)	0.51	3.01	1.50	0.50	6.87	0.12	1.91
Legumes (98 g)	0.12	0.67	2.01	0.21	3.83	0.08	0.49
Pork (117 g)	0.92	4.73	3.30	0.93	14.08	0.23	4.26
Pork (110 g)	0.87	4.44	3.11	0.87	13.23	0.21	4.01
Pork (99 g)	0.78	4.00	2.80	0.79	11.91	0.19	3.61
Pork (92 g)	0.72	3.72	2.60	0.73	11.07	0.18	3.35
Pork (98 g)	0.77	3.96	2.77	0.78	11.79	0.19	3.57

[1]Represents primary food component in mixed dishes.
[2]Skim or 1%.
[3]US $
GHGE, greenhouse gas emissions
CED, cumulative energy demand
WSF, water scarcity footprint

Supplemental Table 5: Mean Healthy Eating Index-2020 components per serving of food, 2011-2018 (n=17,483)

Food category (serving size)[1]	Energy (kcal)	Total fruit (cup eq)	Whole fruit (cup eq)	Total vegetables (cup eq)	Dark green vegetables and legumes (cup eq)	Whole grains (oz eq)	Refined grains (oz eq)	Dairy (cup eq)
				Mean per serving				
Beef (117 g)	244.27	0.00	0.00	0.13	0.02	0.01	0.55	0.10
Poultry (110 g)	215.00	0.00	0.00	0.08	0.01	0.01	0.44	0.02
Seafood (99 g)	174.02	0.00	0.00	0.06	0.00	0.01	0.35	0.02
Eggs (92 g)	170.11	0.00	0.00	0.04	0.01	0.00	0.11	0.09
Legumes (98 g)	145.91	0.00	0.00	0.04	0.46	0.00	0.00	0.00
Pork (117 g)	251.25	0.00	0.00	0.06	0.01	0.01	0.17	0.01
Pork (110 g)	236.22	0.00	0.00	0.06	0.01	0.01	0.16	0.01
Pork (99 g)	212.60	0.00	0.00	0.05	0.01	0.01	0.14	0.01
Pork (92 g)	197.57	0.00	0.00	0.05	0.00	0.01	0.13	0.01
Pork (98 g)	210.45	0.00	0.00	0.05	0.01	0.01	0.14	0.01

[1]Represents primary food component in mixed dishes.

[2]Skim or 1%.

[3]US $

GHGE, greenhouse gas emissions

CED, cumulative energy demand

WSF, water scarcity footprint

Supplemental Table 5 - continued

Food category (serving size)[1]	Total protein foods (oz eq)	Seafood and plant proteins (oz eq)	Unsaturated fats (g)	Saturated fats (g)	Added sugar (tsp)	Sodium (g)
			Mean per serving			
Beef (117 g)	2.06	0.05	6.64	4.97	0.34	520.70
Poultry (110 g)	2.46	0.02	6.88	2.47	0.28	547.88
Seafood (99 g)	2.45	2.40	6.19	1.66	0.13	465.11
Eggs (92 g)	1.52	0.00	7.54	3.90	0.04	366.81
Legumes (98 g)	0.09	1.90	3.57	0.79	0.24	280.90
Pork (117 g)	3.05	0.01	8.43	4.56	0.30	879.07
Pork (110 g)	2.87	0.01	7.93	4.29	0.28	826.48
Pork (99 g)	2.58	0.01	7.14	3.86	0.25	743.83
Pork (92 g)	2.40	0.01	6.63	3.58	0.23	691.24
Pork (98 g)	2.56	0.01	7.06	3.82	0.25	736.32

[1]Represents primary food component in mixed dishes.

[2]Skim or 1%.

[3]US $

GHGE, greenhouse gas emissions

CED, cumulative energy demand

WSF, water scarcity footprint

Supplemental Table 6: Daily per capita servings of protein foods at baseline and after modeled food substitutions, 2011-2018 (n=17,483)

Food category (serving size)[1]	Baseline	Modeled substitutions: 1 serving	2 servings	3 servings
	Mean servings (95% CI)			
Scenario 1				
Beef (117 g)	0.40 (0.38-0.42)	0.23 (0.22-0.25)	0.19 (0.18-0.2)	0.19 (0.18-0.2)
Pork (117 g)	0.19 (0.18-0.2)	0.35 (0.34-0.37)	0.40 (0.38-0.42)	0.40 (0.38-0.42)
Scenario 2				
Poultry (110 g)	0.66 (0.62-0.69)	0.39 (0.37-0.41)	0.29 (0.28-0.31)	0.27 (0.25-0.28)
Pork (110 g)	0.20 (0.19-0.21)	0.47 (0.45-0.48)	0.57 (0.54-0.59)	0.59 (0.57-0.61)
Scenario 3				
Seafood (99 g)	0.19 (0.18-0.21)	0.11 (0.1-0.12)	0.09 (0.08-0.09)	0.08 (0.07-0.09)
Pork (99 g)	0.22 (0.21-0.24)	0.31 (0.29-0.32)	0.33 (0.31-0.35)	0.34 (0.32-0.35)
Scenario 4				
Eggs (92 g)	0.28 (0.26-0.29)	0.14 (0.13-0.15)	0.12 (0.12-0.13)	0.12 (0.11-0.13)
Pork (92 g)	0.24 (0.22-0.26)	0.38 (0.36-0.39)	0.40 (0.38-0.42)	0.40 (0.38-0.42)
Scenario 5				
Legumes (98 g)	0.15 (0.13-0.16)	0.09 (0.08-0.1)	0.08 (0.07-0.08)	0.07 (0.07-0.08)
Pork (98 g)	0.23 (0.21-0.24)	0.28 (0.26-0.3)	0.30 (0.28-0.32)	0.30 (0.28-0.32)

All results were adjusted for energy and survey cycle using linear regression models.

[1]Represents primary food component in mixed dishes.

Supplemental Table 7: Mean daily sustainability impacts at baseline and after modeled replacement of protein foods with pork, 2011-2018 (n=17,483)

Food category (serving size)[1]	GHGE ($kg\ CO_2\ eq$)		CED (MJ)		WSF ($L\ eq \times 10^2$)		Land (m^2)	
	Mean daily per capita impacts (95% CI)							
Baseline	5.09 (5-5.19)		27.39 (26.94-27.84)		36.44 (35.53-37.36)		17.66 (17.08-18.23)	
Beef (117 g)								
1 serving	4.69 (4.61-4.77)	*	26.97 (26.52-27.41)	*	35.29 (34.38-36.21)	*	12.07 (11.66-12.48)	*
2 servings	4.42 (4.34-4.49)	*	26.67 (26.23-27.11)	*	34.48 (33.55-35.41)	*	11.51 (11.12-11.9)	*
3 servings	4.34 (4.27-4.42)	*	26.50 (26.07-26.94)	*	34.10 (33.16-35.03)	*	11.51 (11.12-11.9)	
Poultry (110 g)								
1 serving	5.20 (5.11-5.29)	*	27.73 (27.28-28.18)	*	36.61 (35.7-37.53)	*	17.72 (17.15-18.3)	*
2 servings	5.29 (5.2-5.39)	*	28.01 (27.55-28.46)	*	36.76 (35.84-37.67)	*	17.77 (17.19-18.34)	*
3 servings	5.35 (5.26-5.45)	*	28.19 (27.73-28.65)	*	36.85 (35.94-37.77)	*	17.79 (17.22-18.36)	*
Seafood (99 g)								
1 serving	5.09 (4.99-5.18)	*	26.44 (26.01-26.87)	*	36.59 (35.68-37.51)	*	17.69 (17.11-18.26)	*
2 servings	5.08 (4.99-5.18)	*	25.79 (25.37-26.21)	*	36.71 (35.8-37.63)	*	17.71 (17.13-18.28)	*
3 servings	5.08 (4.99-5.17)	*	25.46 (25.05-25.87)	*	36.78 (35.87-37.7)	*	17.72 (17.14-18.29)	*
Eggs (92 g)								
1 serving	5.12 (5.03-5.22)	*	27.49 (27.04-27.94)	*	36.59 (35.68-37.5)	*	17.69 (17.12-18.27)	*
2 servings	5.13 (5.04-5.23)	*	27.53 (27.08-27.98)	*	36.66 (35.75-37.57)	*	17.71 (17.14-18.28)	*
3 servings	5.14 (5.05-5.23)	*	27.55 (27.1-28)	*	36.69 (35.78-37.6)	*	17.72 (17.14-18.29)	*
Legumes (98 g)								
1 serving	5.13 (5.03-5.22)	*	27.58 (27.13-28.03)	*	36.49 (35.57-37.4)	*	17.70 (17.13-18.28)	*
2 servings	5.16 (5.06-5.25)	*	27.72 (27.26-28.17)	*	36.52 (35.61-37.43)	*	17.76 (17.18-18.33)	
3 servings	5.17 (5.08-5.26)	*	27.79 (27.34-28.24)	*	36.54 (35.62-37.45)	*	17.77 (17.19-18.34)	*

All results were adjusted for energy and survey cycle using linear regression models.

GHGE, greenhouse gas emissions

CED, cumulative energy demand

WSF, water scarcity footprint

HEI-2020, Healthy Eating Index-2020

[1]Represents primary food component in mixed dishes.

*Significantly different than prior modeled substitution with the same column using paired Wald tests at P<0.025 (Bonferonni correction = 0.05 ÷ 2 pairwise tests = 0.025).

Supplemental Table 7 - continued

Food category (serving size)[1]	Fertilizer nutrients (kg × 10^{-3})	Pesticides (kg × 10^{-3})	Diet cost (US $)	Diet quality (HEI-2020 score)
	Mean daily per capita impacts (95% CI)			
Baseline	84.81 (83.72-85.9)	2.37 (2.33-2.42)	16.35 (15.99-16.71)	50.77 (50.19-51.34)
Beef (117 g)				
1 serving	79.49 (78.17-80.82) *	2.10 (2.07-2.13) *	16.31 (15.95-16.67) *	50.73 (50.16-51.31) *
2 servings	76.16 (74.84-77.48) *	1.98 (1.95-2.02) *	16.29 (15.93-16.65) *	50.73 (50.16-51.3)
3 servings	75.39 (74.06-76.72) *	1.97 (1.94-2) *	16.27 (15.91-16.63) *	50.75 (50.18-51.32) *
Poultry (110 g)				
1 serving	85.66 (84.21-87.11)	2.39 (2.35-2.43) *	16.68 (16.31-17.04) *	50.31 (49.73-50.88) *
2 servings	85.98 (84.53-87.44) *	2.40 (2.36-2.44) *	16.95 (16.58-17.32) *	49.99 (49.43-50.56) *
3 servings	86.18 (84.71-87.65) *	2.41 (2.36-2.45) *	17.13 (16.75-17.51) *	49.84 (49.28-50.41) *
Seafood (99 g)				
1 serving	85.22 (84.13-86.31) *	2.38 (2.34-2.42) *	16.33 (15.97-16.69) *	50.54 (49.97-51.11) *
2 servings	85.52 (84.43-86.61) *	2.39 (2.34-2.43) *	16.32 (15.96-16.68) *	50.37 (49.81-50.94) *
3 servings	85.69 (84.61-86.78) *	2.39 (2.35-2.43) *	16.31 (15.95-16.67) *	50.30 (49.73-50.86) *
Eggs (92 g)				
1 serving	85.39 (84.31-86.48) *	2.38 (2.34-2.43) *	16.54 (16.18-16.91) *	50.64 (50.07-51.22) *
2 servings	85.66 (84.59-86.74) *	2.39 (2.35-2.43) *	16.63 (16.27-16.99) *	50.60 (50.02-51.18) *
3 servings	85.77 (84.69-86.85) *	2.39 (2.35-2.43) *	16.67 (16.31-17.03) *	50.59 (50.02-51.17) *
Legumes (98 g)				
1 serving	85.64 (84.25-87.04)	2.38 (2.34-2.43)	16.52 (16.16-16.88) *	50.54 (49.96-51.12) *
2 servings	86.81 (83.99-89.63)	2.40 (2.35-2.44)	16.65 (16.29-17.01) *	50.35 (49.76-50.93) *
3 servings	86.97 (84.16-89.79) *	2.40 (2.35-2.45) *	16.72 (16.36-17.08) *	50.27 (49.69-50.86) *

All results were adjusted for energy and survey cycle using linear regression models.
GHGE, greenhouse gas emissions
CED, cumulative energy demand
WSF, water scarcity footprint
HEI-2020, Healthy Eating Index-2020
[1]Represents primary food component in mixed dishes.
*Significantly different than prior modeled substitution with the same column using paired Wald tests at P<0.025 (Bonferonni correction = 0.05 ÷ 2 pairwise tests = 0.025).

Supplemental Table 8: Mean daily Healthy Eating Index-2020 component scores at baseline and after modeled replacement of protein foods with pork, 2011-2018 (n=17,483)

Food category (serving size)[1]	Total fruit (0-5 points)	Whole fruit (0-5 points)	Total vegetables (0-5 points)	Dark green vegetables and legumes	Whole grains (0-5 points)	Refined grains (0-5 points)
	Mean score (95% CI)					
Baseline	2.05 (1.98-2.12)	2.11 (2.02-2.19)	3.02 (2.97-3.08)	1.57 (1.51-1.63)	2.71 (2.6-2.83)	6.16 (6.07-6.25)
Beef (117 g)						
1 serving	2.05 (1.98-2.12) *	2.11 (2.02-2.19) *	3.01 (2.95-3.06) *	1.57 (1.51-1.63) *	2.71 (2.6-2.83) *	6.24 (6.15-6.33) *
2 servings	2.05 (1.98-2.12) *	2.11 (2.02-2.19) *	2.99 (2.94-3.04) *	1.57 (1.51-1.63) *	2.71 (2.6-2.82) *	6.28 (6.19-6.38) *
3 servings	2.05 (1.98-2.12) *	2.11 (2.02-2.19) *	2.99 (2.93-3.04) *	1.57 (1.51-1.63) *	2.71 (2.6-2.82) *	6.30 (6.21-6.39) *
Poultry (110 g)						
1 serving	2.04 (1.97-2.11) *	2.10 (2.02-2.19) *	3.01 (2.96-3.06) *	1.57 (1.51-1.63) *	2.71 (2.6-2.83) *	6.27 (6.18-6.36) *
2 servings	2.04 (1.97-2.11) *	2.10 (2.02-2.19) *	3.00 (2.95-3.05) *	1.57 (1.51-1.63) *	2.71 (2.59-2.82) *	6.35 (6.26-6.44) *
3 servings	2.04 (1.97-2.11) *	2.10 (2.02-2.18) *	2.99 (2.94-3.04) *	1.57 (1.51-1.63) *	2.71 (2.59-2.82) *	6.39 (6.3-6.49) *
Seafood (99 g)						
1 serving	2.05 (1.98-2.12) *	2.11 (2.02-2.19) *	3.02 (2.97-3.07) *	1.57 (1.51-1.63)	2.71 (2.59-2.82) *	6.19 (6.1-6.28) *
2 servings	2.04 (1.97-2.11) *	2.11 (2.02-2.19) *	3.02 (2.97-3.07) *	1.57 (1.51-1.63)	2.71 (2.59-2.82) *	6.21 (6.12-6.3) *
3 servings	2.04 (1.97-2.11) *	2.11 (2.02-2.19) *	3.02 (2.97-3.07) *	1.57 (1.51-1.63)	2.71 (2.59-2.82) *	6.22 (6.13-6.31) *
Eggs (92 g)						
1 serving	2.05 (1.98-2.12) *	2.11 (2.02-2.19) *	3.02 (2.97-3.08) *	1.57 (1.51-1.63) *	2.71 (2.6-2.83) *	6.17 (6.08-6.26) *
2 servings	2.05 (1.98-2.12) *	2.11 (2.02-2.19) *	3.02 (2.97-3.08)	1.57 (1.51-1.63) *	2.71 (2.6-2.83) *	6.17 (6.08-6.27) *
3 servings	2.05 (1.98-2.11) *	2.11 (2.02-2.19) *	3.02 (2.97-3.08)	1.57 (1.51-1.63)	2.71 (2.6-2.83) *	6.17 (6.08-6.27) *
Legumes (98 g)						
1 serving	2.05 (1.98-2.12) *	2.11 (2.02-2.19) *	2.99 (2.94-3.04) *	1.51 (1.45-1.57) *	2.71 (2.6-2.83) *	6.16 (6.07-6.26) *
2 servings	2.04 (1.97-2.11) *	2.11 (2.02-2.19) *	2.97 (2.92-3.02) *	1.46 (1.4-1.52) *	2.71 (2.6-2.83) *	6.17 (6.07-6.26) *
3 servings	2.04 (1.97-2.11) *	2.10 (2.02-2.19) *	2.96 (2.91-3.01) *	1.44 (1.38-1.5) *	2.71 (2.6-2.82) *	6.17 (6.08-6.26) *

All results were adjusted for energy and survey cycle using linear regression models.

HEI-2020, Healthy Eating Index-2020

[1]Represents primary food component in mixed dishes.

*Significantly different than prior modeled substitution with the same column using paired Wald tests at P<0.025 (Bonferonni correction = 0.05 ÷ 2 pairwise tests = 0.025).

Supplemental Table 8 - continued

Food category (serving size)[1]	Dairy (0-10 points)	Total protein foods (0-5 points)	Seafood and plant proteins (0-5 points)	Unsaturated fats (0-10 points)	Saturated fats (0-10 points)	Added sugar (0-10 points)
	Mean score (95% CI)					
Baseline	5.02 (4.92-5.11)	4.00 (3.96-4.04)	2.37 (2.3-2.43)	4.97 (4.85-5.08)	5.89 (5.79-6)	6.50 (6.38-6.61)
Beef (117 g)						
1 serving	4.97 (4.87-5.06) *	4.04 (4-4.08) *	2.36 (2.3-2.43) *	5.07 (4.96-5.19) *	5.93 (5.82-6.03) *	6.50 (6.39-6.62) *
2 servings	4.94 (4.84-5.03) *	4.05 (4.01-4.09) *	2.36 (2.29-2.43) *	5.14 (5.03-5.25) *	5.95 (5.85-6.05) *	6.51 (6.39-6.62) *
3 servings	4.92 (4.83-5.02) *	4.05 (4.01-4.09)	2.36 (2.29-2.43) *	5.17 (5.06-5.28) *	5.96 (5.86-6.06) *	6.51 (6.39-6.62) *
Poultry (110 g)						
1 serving	5.00 (4.91-5.1) *	4.02 (3.98-4.06) *	2.36 (2.3-2.43) *	4.81 (4.69-4.92) *	5.72 (5.62-5.83) *	6.51 (6.4-6.62) *
2 servings	4.99 (4.89-5.08) *	4.03 (3.99-4.07) *	2.36 (2.29-2.43) *	4.69 (4.58-4.8) *	5.59 (5.48-5.69) *	6.52 (6.41-6.63) *
3 servings	4.98 (4.89-5.08) *	4.03 (4-4.07) *	2.36 (2.29-2.43) *	4.63 (4.52-4.74) *	5.50 (5.4-5.61) *	6.53 (6.41-6.64) *
Seafood (99 g)						
1 serving	5.01 (4.92-5.1) *	4.00 (3.96-4.04) *	2.30 (2.23-2.36) *	4.91 (4.79-5.02) *	5.84 (5.74-5.94) *	6.50 (6.39-6.62) *
2 servings	5.01 (4.91-5.1) *	4.00 (3.96-4.04) *	2.24 (2.18-2.31) *	4.87 (4.75-4.98) *	5.80 (5.69-5.9) *	6.51 (6.39-6.62) *
3 servings	5.00 (4.91-5.1) *	4.00 (3.96-4.04)	2.22 (2.15-2.28) *	4.84 (4.73-4.96) *	5.77 (5.67-5.88) *	6.51 (6.39-6.62) *
Eggs (92 g)						
1 serving	4.97 (4.88-5.07) *	4.02 (3.99-4.06) *	2.37 (2.3-2.43) *	4.95 (4.84-5.07) *	5.93 (5.83-6.04) *	6.50 (6.39-6.61) *
2 servings	4.96 (4.86-5.05) *	4.03 (3.99-4.06) *	2.37 (2.3-2.43) *	4.95 (4.83-5.07) *	5.95 (5.85-6.05) *	6.50 (6.39-6.61)
3 servings	4.95 (4.86-5.05) *	4.03 (3.99-4.06)	2.37 (2.3-2.43) *	4.95 (4.83-5.06) *	5.96 (5.86-6.06) *	6.50 (6.39-6.61)
Legumes (98 g)						
1 serving	5.01 (4.92-5.11) *	4.05 (4.02-4.09) *	2.32 (2.25-2.39) *	4.94 (4.82-5.05) *	5.85 (5.75-5.95) *	6.50 (6.39-6.62) *
2 servings	5.01 (4.92-5.1) *	4.07 (4.03-4.1) *	2.28 (2.21-2.35) *	4.92 (4.8-5.03) *	5.82 (5.71-5.92) *	6.51 (6.4-6.62) *
3 servings	5.01 (4.91-5.1) *	4.07 (4.03-4.1)	2.26 (2.19-2.33) *	4.91 (4.79-5.03) *	5.80 (5.7-5.9) *	6.51 (6.4-6.62) *

All results were adjusted for energy and survey cycle using linear regression models.

HEI-2020, Healthy Eating Index-2020

[1]Represents primary food component in mixed dishes.

*Significantly different than prior modeled substitution with the same column using paired Wald tests at P<0.025 (Bonferonni correction = 0.05 ÷ 2 pairwise tests = 0.025).

Supplemental Table 8 - continued

Food category (serving size)[1]	Sodium (0-10 points)	
	Mean score (95% CI)	
Baseline	4.40 (4.32-4.48)	
Beef (117 g)		
1 serving	4.18 (4.1-4.26)	*
2 servings	4.08 (4-4.16)	*
3 servings	4.06 (3.98-4.13)	*
Poultry (110 g)		
1 serving	4.17 (4.09-4.25)	*
2 servings	4.04 (3.96-4.13)	*
3 servings	4.01 (3.92-4.09)	*
Seafood (99 g)		
1 serving	4.34 (4.26-4.42)	*
2 servings	4.30 (4.22-4.38)	*
3 servings	4.29 (4.21-4.37)	*
Eggs (92 g)		
1 serving	4.26 (4.18-4.34)	*
2 servings	4.22 (4.14-4.3)	*
3 servings	4.21 (4.13-4.29)	*
Legumes (98 g)		
1 serving	4.33 (4.25-4.41)	*
2 servings	4.30 (4.22-4.38)	*
3 servings	4.29 (4.21-4.37)	*

All results were adjusted for energy and survey cycle using linear regression models.
HEI-2020, Healthy Eating Index-2020
[1]Represents primary food component in mixed dishes.
*Significantly different than prior modeled substitution with the same column using paired Wald tests at P<0.025 (Bonferonni correction = 0.05 ÷ 2 pairwise tests = 0.025).

Supplemental Table 9: Mean daily sustainability impacts at baseline and after modeled replacement of protein foods with pork, 2011-2018, based on Recommended Amounts Customarily Consumed (n=17,483)

Food category (serving size)[1]	GHGE (kg CO_2 eq)		CED (MJ)		WSF (L eq × 10^2)		Land (m^2)	
	Mean daily per capita impacts (95% CI)							
Baseline	5.09 (5-5.19)		27.39 (26.94-27.84)		36.44 (35.53-37.36)		17.66 (17.08-18.23)	
Beef (90 g)								
1 serving	4.71 (4.63-4.79)	*	26.98 (26.53-27.42)	*	35.35 (34.44-36.26)	*	11.82 (11.43-12.21)	*
2 servings	4.35 (4.28-4.43)	*	26.59 (26.14-27.03)	*	34.31 (33.38-35.25)	*	10.59 (10.21-10.96)	*
3 servings	4.19 (4.11-4.27)	*	26.33 (25.89-26.76)	*	33.68 (32.72-34.63)	*	10.59 (10.21-10.98)	
Poultry (91 g)								
1 serving	5.19 (5.09-5.28)	*	27.66 (27.21-28.11)	*	36.58 (35.66-37.49)	*	17.86 (17.28-18.45)	*
2 servings	5.28 (5.18-5.37)	*	27.93 (27.48-28.38)	*	36.71 (35.79-37.62)	*	18.09 (17.48-18.7)	*
3 servings	5.34 (5.25-5.44)	*	28.13 (27.67-28.58)	*	36.80 (35.89-37.72)	*	18.29 (17.61-18.96)	*
Seafood (89 g)								
1 serving	5.09 (4.99-5.18)	*	26.49 (26.06-26.93)	*	36.58 (35.67-37.5)	*	17.78 (17.2-18.35)	*
2 servings	5.08 (4.99-5.18)	*	25.81 (25.38-26.23)	*	36.70 (35.79-37.62)	*	17.92 (17.31-18.52)	
3 servings	5.08 (4.98-5.17)	*	25.45 (25.04-25.85)	*	36.78 (35.86-37.69)	*	18.07 (17.36-18.78)	
Eggs (82 g)								
1 serving	5.13 (5.03-5.22)	*	27.52 (27.07-27.97)	*	36.61 (35.7-37.52)	*	17.85 (17.28-18.43)	*
2 servings	5.15 (5.05-5.24)	*	27.59 (27.14-28.04)	*	36.70 (35.79-37.61)	*	17.92 (17.34-18.49)	*
3 servings	5.16 (5.06-5.25)	*	27.63 (27.18-28.08)	*	36.74 (35.84-37.65)	*	17.94 (17.37-18.52)	*
Legumes (96 g)								
1 serving	5.13 (5.03-5.22)	*	27.56 (27.11-28.01)	*	36.47 (35.56-37.39)	*	17.72 (17.14-18.3)	*
2 servings	5.15 (5.06-5.25)	*	27.69 (27.24-28.15)	*	36.50 (35.59-37.41)	*	17.78 (17.19-18.36)	*
3 servings	5.17 (5.07-5.26)	*	27.76 (27.31-28.21)	*	36.51 (35.6-37.43)	*	17.80 (17.22-18.38)	*

All results were adjusted for energy and survey cycle using linear regression models.
Serving size of pork is 89 g.
GHGE, greenhouse gas emissions
CED, cumulative energy demand
WSF, water scarcity footprint
HEI-2020, Healthy Eating Index-2020
[1]Represents primary food component in mixed dishes.
*Significantly different than prior modeled substitution with the same column using paired Wald tests at P<0.025 (Bonferonni correction = 0.05 ÷ 2 pairwise tests = 0.025).

Supplemental Table 9 - continued

Food category (serving size)[1]	Fertilizer nutrients ($kg \times 10^{-3}$)		Pesticides ($kg \times 10^{-3}$)		Diet cost (US $)		Diet quality (HEI-2020 score)	
Baseline	84.81 (83.72-85.9)		2.37 (2.33-2.42)		16.35 (15.99-16.71)		50.77 (50.19-51.34)	
Beef (90 g)								
1 serving	79.56 (78.11-81.01)	*	2.02 (1.98-2.05)	*	16.30 (15.94-16.66)	*	50.74 (50.17-51.32)	*
2 servings	75.22 (72.72-77.71)	*	1.84 (1.78-1.89)	*	16.26 (15.9-16.62)	*	50.74 (50.16-51.31)	
3 servings	74.60 (72.03-77.16)	*	1.83 (1.78-1.89)		16.23 (15.87-16.59)	*	50.76 (50.19-51.33)	*
Poultry (91 g)								
1 serving	88.33 (86.45-90.22)	*	2.43 (2.38-2.47)	*	16.62 (16.25-16.98)	*	50.35 (49.78-50.93)	*
2 servings	91.85 (88.78-94.93)	*	2.48 (2.42-2.55)	*	16.88 (16.51-17.25)	*	50.01 (49.44-50.58)	*
3 servings	94.63 (89.64-99.62)	*	2.53 (2.43-2.62)	*	17.08 (16.7-17.45)	*	49.81 (49.24-50.38)	*
Seafood (89 g)								
1 serving	86.64 (84.93-88.34)	*	2.40 (2.36-2.45)	*	16.33 (15.97-16.69)	*	50.55 (49.98-51.12)	*
2 servings	88.76 (84.94-92.58)		2.44 (2.37-2.51)		16.31 (15.95-16.67)	*	50.38 (49.81-50.94)	*
3 servings	91.11 (83.87-98.34)		2.48 (2.35-2.6)		16.30 (15.94-16.66)	*	50.29 (49.72-50.86)	*
Eggs (82 g)								
1 serving	88.09 (86.23-89.95)	*	2.42 (2.37-2.47)	*	16.57 (16.2-16.93)	*	50.64 (50.06-51.21)	*
2 servings	89.10 (87.2-90.99)	*	2.44 (2.39-2.49)	*	16.69 (16.32-17.05)	*	50.58 (50.01-51.16)	*
3 servings	89.51 (87.6-91.42)	*	2.45 (2.4-2.49)	*	16.74 (16.38-17.11)	*	50.57 (50-51.15)	*
Legumes (96 g)								
1 serving	85.89 (84.71-87.07)	*	2.39 (2.35-2.43)	*	16.51 (16.14-16.87)	*	50.55 (49.97-51.13)	*
2 servings	86.87 (85.36-88.39)	*	2.40 (2.36-2.45)	*	16.63 (16.27-16.99)	*	50.34 (49.76-50.92)	*
3 servings	87.28 (85.71-88.86)	*	2.41 (2.36-2.45)	*	16.70 (16.33-17.06)	*	50.26 (49.68-50.85)	*

All results were adjusted for energy and survey cycle using linear regression models.
Serving size of pork is 89 g.
GHGE, greenhouse gas emissions
CED, cumulative energy demand
WSF, water scarcity footprint
HEI-2020, Healthy Eating Index-2020
[1]Represents primary food component in mixed dishes.
*Significantly different than prior modeled substitution with the same column using paired Wald tests at $P<0.025$ (Bonferonni correction = 0.05 ÷ 2 pairwise tests = 0.025).

Supplemental Figure 1: Mean percent change Healthy Eating Index-2020 component scores after modeled replacement of protein foods with pork, 2011-2018 (n=17,483)

Food category[1]	Number of serving substitutions	Total fruit	Whole fruit	Total veg.	Dark green veg. and legumes	Whole grains	Refined grains	Dairy	Total protein foods	Seafood and plant proteins	Unsat. fats	Sat. fats	Added sugar	Sodium
		Percent change in HEI-2020 component score from baseline												
Beef (117 g)	1 serving	0.0%	0.0%	-0.7%	-0.1%	-0.1%	1.3%	-1.0%	1.0%	-0.1%	2.2%	0.5%	0.1%	-5.1%
	2 servings	0.0%	0.0%	-1.1%	-0.2%	-0.1%	2.0%	-1.6%	1.3%	-0.1%	3.5%	0.9%	0.1%	-7.4%
	3 servings	0.0%	0.0%	-1.4%	-0.3%	-0.1%	2.3%	-1.9%	1.3%	-0.2%	4.2%	1.0%	0.2%	-7.9%
Poultry (110 g)	1 serving	-0.2%	-0.1%	-0.5%	-0.1%	-0.1%	1.7%	-0.3%	0.6%	-0.1%	-3.1%	-2.9%	0.2%	-5.4%
	2 servings	-0.3%	-0.2%	-0.9%	-0.3%	-0.2%	3.1%	-0.6%	0.9%	-0.2%	-5.4%	-5.3%	0.4%	-8.4%
	3 servings	-0.4%	-0.2%	-1.2%	-0.4%	-0.2%	3.8%	-0.7%	0.9%	-0.3%	-6.7%	-6.7%	0.5%	-9.3%
Seafood (99 g)	1 serving	-0.1%	0.0%	-0.1%	0.0%	-0.2%	0.5%	-0.1%	0.0%	-2.6%	-1.1%	-0.9%	0.1%	-1.5%
	2 servings	-0.1%	-0.1%	-0.2%	0.0%	-0.3%	0.8%	-0.2%	0.0%	-5.1%	-2.0%	-1.7%	0.1%	-2.4%
	3 servings	-0.2%	-0.1%	-0.3%	0.0%	-0.3%	1.0%	-0.3%	0.0%	-6.2%	-2.5%	-2.1%	0.1%	-2.7%
Eggs (92 g)	1 serving	-0.1%	0.0%	0.0%	0.0%	0.0%	0.2%	-0.9%	0.6%	0.0%	-0.2%	0.7%	0.0%	-3.4%
	2 servings	-0.1%	0.0%	0.0%	0.0%	0.0%	0.2%	-1.2%	0.8%	0.0%	-0.3%	0.9%	0.0%	-4.3%
	3 servings	-0.1%	-0.1%	0.0%	0.0%	0.0%	0.2%	-1.4%	0.8%	0.0%	-0.3%	1.0%	0.0%	-4.4%
Legumes (98 g)	1 serving	-0.1%	0.0%	-1.2%	-4.3%	-0.1%	0.1%	-0.1%	1.4%	-1.8%	-0.6%	-0.7%	0.1%	-1.6%
	2 servings	-0.2%	-0.1%	-1.9%	-7.6%	-0.1%	0.1%	-0.1%	1.7%	-3.8%	-1.0%	-1.3%	0.2%	-2.4%
	3 servings	-0.2%	-0.1%	-2.1%	-8.7%	-0.1%	0.1%	-0.2%	1.7%	-4.5%	-1.1%	-1.6%	0.2%	-2.6%

All results were adjusted for energy and survey cycle using linear regression models.
HEI-2020, Healthy Eating Index-2020
[1]Represents primary food component in mixed dishes.

Supplemental Document 1: Foodprint 2.0 model structure and formulas

This was originally published in Conrad et al. (2024). Foodprint 2.0: A computational simulation model that estimates the agricultural resource requirements of diet patterns. PLOS ONE 2024;19(9):e0306097. It has been reproduced and modified for the present article with permission.

Model structure

Foodprint 2.0 is a spreadsheet model that estimates the amount of agricultural land, fertilizer nutrients (nitrogen, phosphorus-P_2O_5, potash-K_2O, and sulfur), pesticides (sum of herbicides, insecticides, and fungicides), and irrigation water needed to meet user-defined diet patterns in the US. These output data are provided for each of the 208 foods and 10 food categories included in the model. The model contains 20 integrated worksheets that use embedded data and calculations (**Figure 1**). Users enter dietary data into the *Input* worksheet and the embedded computations in subsequent worksheets transform the mass quantity of these foods in stepwise fashion as they move backwards through the food system from being consumer foods to processed products to agricultural commodities, and ultimately to the agricultural resources needed to produce these commodities. Each of these worksheets is described below with a focus on new features added to the model, and readers should refer to the original Foodprint documentation(1) for a detailed description of the underlying computations. All model data represent the mean of 2009-2018 to align with contemporary dietary data from the NHANES unless otherwise noted in the model, but users are not limited to these data years. Supplemental documentation can be found

elsewhere, which include readme files, analytic code, step-by-step instructions, and supplemental data for 1999-2021 (https://doi.org/10.17026/dans-zmh-tzn3). Data in all worksheets represent the US national food system so users interested in modeling other geopolitical scales should use data relevant to their area of interest.

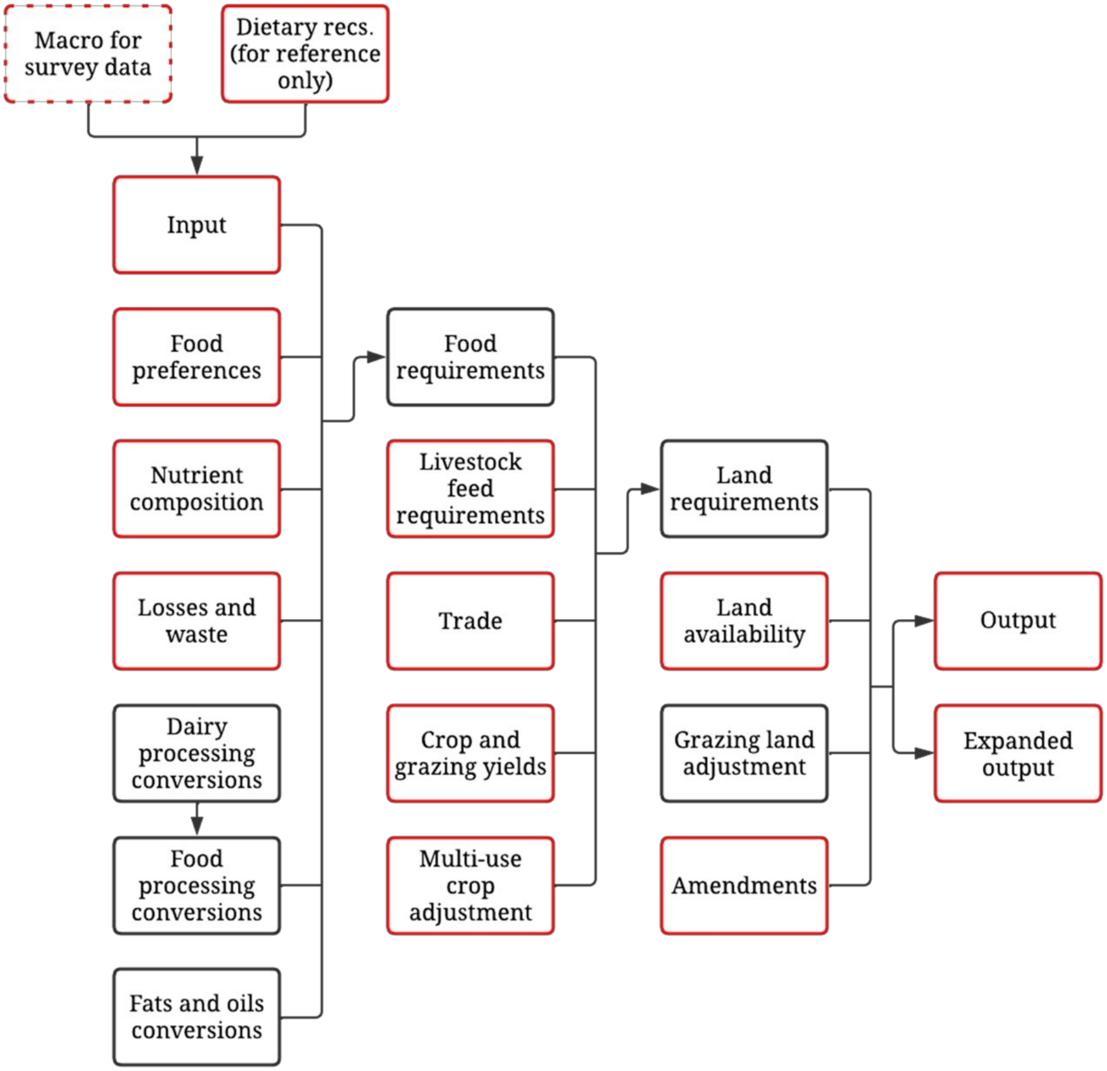

Fig 1: Structure of Foodprint 2.0

Each box represents a distinct worksheet in the model. Those with a dashed border represent an embedded macro and those with a red border represent worksheets with new features or updated data.

Foodprint 2.0 and supporting documents can be accessed at: https://doi.org/10.17026/dans-zmh-tzn3

Input

Users begin by entering information on the population size and trade system (open or closed) of interest. An open trade system is one in which agricultural commodities are imported into and exported out of the study area, which can be useful for evaluating the agricultural resource requirements of food demand in the US, which includes the resources associated with imported food and excludes the resources associated with exported foods. The model accounts for this by incorporating data on the agricultural trade balance of each commodity from the *Trade* worksheet, which is discussed below. A closed system does not account for trade and can be useful for evaluating the capacity for a given food system (at any geographic scale) to feed their populations with food produced within the study area.(2) Data from the *Input* worksheet are used by the *Food requirements* worksheet, which is discussed below.

The *Input* worksheet accepts user-inputted data on the daily intake of 22 food groups: grains; dark green vegetables; red and orange vegetables; dry beans, lentils, and peas; starchy vegetables; other vegetables; fluid milk and yogurt; cheese and other dairy; soy milk; nuts; tofu; beef; pork; chicken; turkey; eggs; aquatic food; plant oils; dairy fats; lard and tallow; and

sweeteners. Users have two options for entering these data. First, these data can be entered manually as per capita intakes, which was the original design of the model. Users can acquire data on actual per capita intakes from the US Department of Agriculture's Loss-Adjusted Food Availability (LAFA) data series,(3) or users can enter theoretical dietary data which can be useful for investigating counterfactual scenarios.(1)

Second, users can use a new embedded macro to automate the input of individual-level dietary data from surveys, such as the NHANES or others. Supplemental documentation provides Stata programming code and step-by-step instructions that describe how to prepare the diet input file using NHANES dietary data (https://doi.org/10.17026/dans-zmh-tzn3). This documentation also describes how to use supplemental databases including the USDA Food patterns Equivalents Database(4) and Food and Nutrient Database for Dietary Studies(5) to convert dietary data from mass quantity in NHANES into servings of each food group needed by the model. The macro will iteratively enter each participant's dietary data into the *Input* worksheet and export the results into a separate file that users can import into their preferred statistical programming package for analysis.

Dietary recommendations

The calculations in the *Dietary recommendations* worksheet adjust the recommended intake of food groups from the Healthy US Style Dietary Pattern in the 2020-2025 Dietary Guidelines for Americans(6) to the age-sex distribution of the population of interest (the original model used the MyPyramid recommendations). Data from this worksheet are not used by the model's embedded

computations but can be used as a reference in case users want to manually input dietary data that align with recommended intakes into the *Input* worksheet.

Food preferences

Dietary data are input into the model on the basis of food groups rather than individual foods, so the *Food preferences* worksheet estimates the individual foods that comprise each food group (e.g., dark green leafy vegetables includes spinach and kale) using data on per capita food availability from LAFA.(3) This worksheet allows users to indicate whether a food can be produced within the study area. Foods that cannot be produced within the study area will not be included in the model computations, and the consumption amount for that food will be reapportioned to other foods within that food group in proportion to the per capita consumption amounts of the other foods within that food group. These data are used by the *Food requirements* worksheet.

Nutrient composition

The *Nutrient composition* worksheet tabulates data on serving sizes (grams per serving) of each food, as well as their content of energy and macronutrients, which were acquired from USDA FoodData Central(7) (the original model used data from USDA Nutrient Database for Standard Reference, which has since been replaced with updated data from FoodData Central). Data on serving sizes are linked to the *Food requirements* worksheet where they are used to convert the servings (ounce equivalents or cup equivalents) of each food consumed to the equivalent mass

quantity of agricultural commodities. Data on the nutrient content of each food are linked to the *Output* worksheet where they are used to estimate the energy and macronutrient content of the user-defined diet pattern on a per capita basis, but users interested in estimating individual-level nutrient intakes are recommended to use the nutrient content information from their source data (e.g., NHANES).

Losses and waste

Data on retail loss, inedible portions, cooking loss, and consumer waste for each food are tabulated in the *Losses and waste* worksheet. These data were acquired from the most recent LAFA(3) (the original model used data from earlier LAFA versions). These data are used by the *Food requirements* worksheet to convert the amount of food consumed to the corresponding amount of agricultural commodities needed.

Dairy processing

The *Dairy processing conversions* worksheet uses data on the amount of fat and non-fat solids in dairy foods from the USDA Economic Research Service (ERS)(8) and FoodData Central(7) to estimate the total fluid milk requirement in a given diet. Two calculations are used to estimate total fluid milk equivalents using data on fat and non-fat solids, and the larger estimate represents the limiting dairy fraction (i.e., fat solids or non-fat solids) that is used by the *Processing conversions* worksheet. The total fluid milk equivalent (*FM*) based on dairy fat solids (*f*) in a given diet can be expressed as:

$$FM_{fat} = \sum_{i=1}^{N_D} (Food\ Intake_i \ \times Proportion\ of\ fat\ solids_i)/0.037,$$

where *Food intake* is the annual per capita consumption amount of each dairy food (*i*), *Proportion of fat solids* is the mass quantity of dairy fat solids in each dairy food as a proportion of its total mass quantity, N_D is the total number of dairy foods included, and the constant 0.037 is the mass quantity of fat solids in fluid milk as a proportion of its total mass quantity.

The total fluid milk equivalent based on dairy non-fat solids (*n*) in a given diet can be expressed as:

$$FM_{non-fat} = \sum_{i=1}^{N_D} (Food\ Intake_i \ \times Proportion\ of\ non\text{-}fat\ solids_i)/0.086,$$

where the constant 0.086 is the mass quantity of non-fat solids in fluid milk as a proportion of its total mass weight.

Food processing

The *Processing conversions* worksheet estimates coefficients that convert the mass quantity of foods to agricultural commodities, which accounts for losses that occur from processing the raw agricultural commodities into consumer foods. These data were acquired from the USDA ERS(8) and personal communication with experts, as documented in the model and elsewhere.(1) These

data are used by the *Food requirements* worksheet to convert the amount of food consumed to the corresponding amount of agricultural commodities needed.

Fat and oil processing

The *Fat and oil conversions* worksheet estimates the amount of individual fats and oils in processed food products that typically contain multiple types of fats and oils: salad dressing, cooking oil, margarine, shortening, and other edible fats and oils. These data were acquired from the USDA ERS.(8, 9) These data are used by the *Land requirements* worksheet to convert the amount of food consumed to the corresponding amount of agricultural commodities needed.

Food requirements

The *Food requirements* worksheet uses data on food intake, serving sizes, food preferences, losses and waste, and processing conversions from prior worksheets to convert data on servings of food intake to the equivalent mass quantity of agricultural commodities per year (lb/person/y). This is expressed as:

$$Agricultural\ commodity_i = (Food\ intake_i \times Serving\ size_i)/(454\ g/lb) \times$$
$$Food\ preference_i \times Loss\ and\ waste_i \times Processing\ conversion_i \times 365\ d/y,$$

where *Food intake* is the number of servings consumed per capita per day of each food (*i*), *Serving size* (grams/serving) is a coefficient that converts the serving units (cup-equivalents,

ounce-equivalents, or teaspoons) of each food to grams, *Food preference* is the adjusted preference of a given food relative to other foods within that food group, *Loss and waste* is a coefficient that adjusts the amount of each food in its as-consumed form to its equivalent gram amount before losses and waste that occur during the retail and consumer stages of the food system, and *Processing conversion* is a coefficient that converts the amount of each food to the equivalent pounds of agricultural commodity.

Livestock feed requirements

Data in this worksheet report feed conversions that represent the amount of agricultural commodities needed to produce animal products including beef, pork, poultry, dairy, eggs, and farmed aquatic food. These values are used by the *Land requirements* worksheet to estimate the amount of agricultural land needed to produce agricultural commodities.

Feed conversions for beef cattle, dairy cattle used for beef, dairy cattle used for dairy, swine, layers, broilers, and turkeys were acquired from a separate computational simulation model developed by Peters et al.(10) The feed requirements model represents the stocks and flows of each livestock category that includes production animals (e.g., mature productive cows) and support animals (e.g., dry cows and replacement heifers). The nutritional requirements for each life stage were acquired from the National Research Council.(11) A simplified list of the most common feed ingredients included in the model were corn grain, corn silage, soybean meal, hay, haylage, and grazed forage, and their nutrient contents were acquired from DairyOne.(12) Feed

rations were balanced to meet energy and crude protein requirements for each livestock category for each life stage. Further details can be found elsewhere.(10)

Feed conversions for farmed aquatic food are a new feature to Foodprint 2.0. Aquatic foods from capture fisheries are included in the dietary data entered into the *Input* worksheet but this fraction is not used to estimate agricultural resource requirements because these foods do not rely on agricultural production. Six species that represent 83% of farmed aquatic food consumption in the US(13) were included in the model: carp, catfish, salmon, shrimp, tilapia, and trout. For each species, the share of consumption from farmed versus wild-caught systems was acquired from Kroetz et al., 2020.(14) The nutritional requirements (*f*: energy and crude protein) for each species (s) were estimated as:

$$\begin{aligned} Nutritional\ requirements_{sf} &= Feed\ conversion\ ratio_s \times Processing\ efficiency_s \times Feed\ ration_{sf} \\ &\times Nutrient\ content_f, \end{aligned}$$

where *Feed conversion ratio* is the total weight of feed administered over the lifetime of a species divided by the weight gained(15), *Processing efficiency* is the mass quantity of edible fish per mass quantity of live fish, *Feed ration* is the mass quantity of energy or crude protein needed to produce a given mass quantity of edible fish from MacLeod et al, 2020.(15), and *Nutrient content* is the amount of energy or crude protein per mass quantity of each ingredient from USDA FoodData Central,(7) DairyOne,(12) Feedipedia,(16) and Peters et al., 2014(10).

Foodprint 2.0 uses corn and soybean meal to satisfy nutritional requirements for each farmed aquatic food species, but users can incorporate additional feed ingredients as needed.

Trade

Accounting for food trade is a new feature in Foodprint 2.0. Users can indicate whether to model an open or closed food system in the *Input* worksheet, which dictates whether data in the *Trade* worksheet will be incorporated into the model's calculations. The *Trade* worksheet calculates the trade balance for each food using data on beginning stocks, imports, and exports from the USDA ERS Food Availability data series(17) and USDA Foreign Agricultural Service's Global Agricultural Trade System.(18) The annual trade balance for each food (i) is expressed as:

$$Trade\ balance_i = Beginning\ stock_i + Import_i + Export_i,$$

where *Beginning stock* is the mass quantity of a given food remaining from the previous year, *Import* is the mass quantity of a given food imported into the study area, and *Export* is the mass quantity of a given food exported from the study area. The trade balance for each food is used by the *Land requirements* worksheet to adjust the amount of agricultural commodities needed for a given diet pattern. A positive trade balance indicates that fewer agricultural commodities are needed for a given diet pattern, and a negative trade balance indicates that additional agricultural commodities are needed.

Crop and grazing yields

The *Crop and grazing yields* worksheet compiles data on the amount of each crop (and grazing land) produced per acre harvested (in units reported from original sources) and converts them to pounds per acre. These data are used by the *Land requirements* worksheet, and all data were updated from the original version of the model. Data on crop yields were acquired from USDA National Agricultural Statistics Service(19) and unit conversions (from volume or metric units to lbs.) were acquired from USDA ERS.(8) USDA does not report yields from grazing lands (cropland pasture and permanent pasture) so these were estimated using information on the nutrient composition of pasture, total mass quantity of pasture consumed by livestock, and total land area of pasture used for grazing from multiple sources,(20-22) as described elsewhere.(1)

Multiuse crops

Some crops are used to produce multiple end products, such as meal and oil from corn, so taking a simple sum of the land requirements of each end product will lead to an overestimation of total land requirements. The *Multiuse crop adjustment* worksheet prevents this double counting by deriving coefficients (multiuse crop adjustments) that represent the potential land savings attributable to multiuse crops, which are used by the calculations embedded in the *Land requirements* worksheet. This is performed in eight steps which are described below, and all calculations are shown in the worksheet. This worksheet was modified in Foodprint 2.0 to account for the multiuse crops used to feed farmed aquatic food species.

Step 1 of the multiuse crop adjustment estimates the amount of protein from corn gluten used as livestock feed that is co-produced from wet milling whole corn for starch and sweeteners (high fructose corn syrup, glucose, and dextrose) for human consumption. These calculations use data on the amount of corn (as an agricultural commodity) needed to produce a given amount of each type of corn sweetener for human consumption (from the *Food requirements* worksheet), the amount of oil produced from a given amount of corn (from the *Fat and oil conversions* worksheet), the amount of livestock feed produced from a given amount of corn, and the protein content of livestock feed.(8, 23)

Step 2 estimates the amount of oil from canola, olives, peanuts, and soybeans produced for human consumption. These calculations use data on the amount of agricultural land needed to produce these crops and their oil content from the *Food requirements* and *Fat and oil conversions* worksheets. Step 3 estimates the amount of protein from soybean meal used as a livestock feed that is co-produced from crushing whole soybeans for oil, which uses data on the amount of each product consumed (from the *Input* worksheet) and the protein content of feeds (from the *Livestock feed* requirements worksheet). This step also estimates the amount of lard and tallow produced from the co-production of each animal-based food for human consumption.

Step 4 computes the multiuse crop adjustment for land used to grow corn by estimating the total amount of corn oil consumed (from the *Food requirements* and *Fat and oil conversions* worksheets) and the amount of corn oil spared (the lowest value between the total amount of corn oil consumed and the total amount of corn oil produced as a co-product of sweetener production from Step 1). Step 5 estimates the remaining amount of lard and tallow needed to

satisfy dietary demands (from the *Food requirements* and *Fat and oil conversions* worksheets) after subtracting the total amount of lard and tallow coproduced from the processing of animal-based foods for human consumption (from Steps 1 and 3). Step 6 computes the multiuse crop adjustment for land used to grow oilseeds by accounting for the amount that can be offset by surplus corn oil (from Steps 1, 2, and 4), lard, and tallow (from Step 5). Step 7 estimates the amount of protein for livestock feed coproduced from the processing of soybeans and oilseeds using data from Step 2, the *Fat and oil conversions* worksheet, and USDA ERS.(8) Step 8 computes the multiuse crop adjustment for land used to grow soybeans using data on the coproduction of protein for livestock feed from oilseeds (from Step 7) and corn (from Step 1), and the total protein content of livestock feeds (from Step 3). The data obtained from Steps 4, 6, and 8 (corn and oilseeds spared) is then used to adjust the calculation of land use for these crops in the *Land requirements* worksheet.

Land requirements

The *Land requirements* worksheet estimates the acres of each type of land (cultivated cropland, cropland pasture, and permanent pasture) needed to meet dietary demands for each food in a given diet. For all foods except fats and oils, this is expressed as:

$$Required\ land_{il} = [Agricultural\ commodity_i - (Trade\ balance_i / Population)] / Yield_{il},$$

For fats and oils, this is expressed as:

$$Required\ land_{il} = (Agricultural\ commodity_i - Trade\ balance_i / Population) \times Fat\ and\ oil\ processing\ conversion_i \times Multiuse\ crop\ adjustment_i / Yield_{il}$$

where *Required land* (acres/person/yr) represents the amount of each type of land (*l*) needed to produce each food (i), *Agricultural commodity* is the quantity of each agricultural commodity needed to meet the demand for each food (from the *Food requirements* worksheet) in lbs/yr, *Trade balance* is the total trade balance for each food per year (from the *Trade* worksheet), *Population* is the number of individuals in the study area (from the *Input* worksheet), *Fat and oil processing conversion* is the amount of fats and oils in processed foods, *Multiuse crop adjustment* is an adjustment factor to account for the potential land savings from crops that are used to produce multiple end products, and *Yield* represents the mass quantity of each crop produced per unit of land area for each type of land (lb/acre).

Amendments

A new feature of the model allows users to estimate the amount of fertilizer nutrients (nitrogen, phosphorus-P_2O_5, potash-K_2O, and sulfur), pesticides (sum of herbicides, insecticides, and fungicides), and irrigation water needed to meet dietary demands. Data on the amount of fertilizer nutrients and pesticides applied to each crop were collected from the USDA Census of Agriculture(24, 25) and the USDA Agricultural Surveys.(26, 27) The Census of Agriculture collects this information from approximately 1.5 million operations with >$1,000 of sales every

five years, and data on an additional 500,000 operations is acquired through imputation and calibration. Participation is required by federal law, and data are collected by mail, internet, telephone, and in person.(24, 25) Agricultural Surveys collect data from 65,000-81,000 producers annually, mostly by phone and the remainder are collected by mail and in person.(26, 27) Data on the amount of irrigation water applied to each crop were collected from the USDA Irrigation and Water Management Surveys.(28) All producers who indicated irrigation activity in the Census of Agriculture are contacted by mail every five years, and surveys are submitted by mail, online, telephone, or in person. Approximately 35,000 producers are surveyed and data gaps are filled by statistical imputation and calibration.(28)

The *Amendments* worksheet tabulates the mean annual quantity of each amendment applied per unit of land area for each agricultural commodity, adjusted for the proportion of land on which the amendments were applied. These data are used by the *Output* and *Expanded output* worksheets. These values do not include the on-farm resources applied directly to livestock, such as insecticide treatments.

Land availability

Not all land in farms is suitable for agricultural production, and not all agriculturally productive land is equally suitable for the production of all agricultural commodities. The *Land availability* worksheet performs a series of calculations to estimate the quantity of land suitable for the production of cultivated crops, perennial forages, and grazing, using data acquired from USDA National Agricultural Statistics Service(19) and USDA ERS.(21, 29) The first calculation

estimates a coefficient for *Cropping intensity*, which accounts for cases in which multiple crops are produced from the same cropland (otherwise, a simple sum of land area currently dedicated to cultivating each crop would overestimate total cropland). This is expressed as:

$$Cropping\ intensity = \frac{Area\ harvested\ for\ (field\ crops + vegetables + \ other\ cultivated\ crops)}{Total\ area\ in\ cultivated\ crops}$$

Total area in cultivated crops excludes the area in farm buildings, roads, rock outcrops, waterways, and wetlands. The area in productive cropland is estimated by:

$$Productive\ cropland = Cropland\ harvested + Cropland\ used\ only\ for\ pasture\ and\ grazing$$

and the *Proportion of productive land cultivated* is estimated by:

$$Proportion\ of\ productive\ land\ cultivated = Total\ area\ of\ cultivated\ crops\ harvested / Productive\ cropland,$$

where the *Total area of cultivated crops harvested* excludes area on which hay, forage, grass, Christmas trees or short rotation woody crops were harvested. Available cultivated cropland is estimated by:

$$Available\ cultivated\ food\ cropland$$
$$= Productive\ cropland \times Percentage\ of\ productive\ land\ cultivated$$
$$/\ Cropping\ intensity - \ Non\text{-}food\ cropland\ area$$

where *Non-food cropland area* is the total number of acres harvested of cotton, tobacco, and nursery crops. Available grazing land is estimated by:

$$Available\ grazing\ land$$
$$= Available\ permanent\ pasture + Available\ woodland\ used\ for\ grazing,$$

where *Available permanent pasture* includes land that is used for the production of native (range) and seeded (pasture) forage species that are consumed directly by livestock rather than being cultivated for hay, and *Available woodland used for grazing* represents land that consists mainly of forest that have grass and other forage growth that is used for livestock grazing.

Grazing land adjustment

Livestock are grazed on land that is generally not suitable for crop production but can also be grazed on cropland when it is available. The calculations in the *Grazing land adjustment* worksheet estimate the amount of cropland that can be used for grazing if all land used for permanent pasture has been used and there is a remaining need for grazing land to meet dietary demands. In the first step, the ratio of available grazing land to available cropland (*RA*) is expressed as:

$$RA = Available\ grazing\ land / Available\ cropland,$$

from the *Land availability* worksheet. In the second step, the ratio of required grazing land to required cropland (*RR*) is expressed as:

$$RR = \frac{\sum_{i=1}^{N} Required\ grazing\ land_i}{\sum_{i=1}^{N} (Required\ Cropland_i / Cropping\ intensity + Required\ forage\ land_i)}$$

where *Required grazing land* is the quantity of grazing land needed to produce each food (*i*), *Required cropland* is the quantity of all cropland needed to meet dietary demands, *Required forage land* is the quantity of land used for forages needed to meet dietary demands from the *Land requirements* worksheet, and *N* is the total number of foods for which data are available. The third step estimates the ratio of grazing yield from grazing land to grazing yield from cropland (*RY*) as:

$$RY = Yield_g / Yield_p,$$

where *Yield* represents the mass quantity of grazed forage harvested by livestock per acre on land used for grazing (*g*) and all cropland (*p*). The fourth step calculates two values that are used by the *Output* and *Expanded output* worksheets. The amount of cropland that is used for grazing is solved by:

$$If\ RR > RA, then\ Cropland\ used\ for\ grazing$$

$$= \frac{[\sum_{i=1}^{N} Required\ grazing\ land - RA \times \sum_{i=1}^{N} (Required\ Cropland_i / Cropping\ intensity + Required\ for \text{[illegible]}}{RA + RY}$$

otherwise, *Cropland used for grazing* = 0.

and the grazing land offset by cropland grazed is calculated as:

$$Grazing\ land\ offset = Cropland\ used\ for\ grazing \times RY.$$

Output

The *Output* worksheet presents summary results of the proportion of agricultural land used by land type (cultivated cropland, total cropland, and grazing land), energy and macronutrient intake, and the amount of agricultural resources (land, fertilizer nutrients, pesticides, and irrigation water) associated with 10 land types (grains, vegetables, fruit, pulses, nuts, feed grains and oilseeds, sweeteners, hay, cropland pasture, and grazing land). Summary results for the use of fertilizer nutrients, pesticides, and irrigation water are a new feature of the model, and these data are also presented visually as pie charts.

The *Output* worksheet also reports the proportion of the population that can be fed a given diet with current resource use efficiencies, which can be used in conjunction with the closed trade system to answer questions about the capacity to localize food production at sub-national scales. It is not recommended that users estimate the proportion of the population fed in an open trade

system because the formula used to obtain this value is dependent on a fixed land base in the US which is not relevant when food is imported from outside the US. The number of people that can be fed a given diet is constrained by the land type that is most limited (cultivated cropland, all cropland, and all productive land). The number of people that can be fed (*Population fed*) on each land type is solved by:

$$Population\ fed_{cultivated\ cropland} = Available\ cultivated\ food\ cropland \Big/ \sum_{i=1}^{N_g} Required\ cultivated\ cropland_i$$

$$Population\ fed_{\ all\ cropland} = \frac{Available\ food\ cropland, all\ uses}{\sum_{i=1}^{N_g}(Required\ cultivated\ cropland\ _i / Cropping\ intensity + Required\ perennial\ cropland_i) + Cropland\ used\ for\ grazing}$$

$$Population\ fed_{all\ productive\ land} = \frac{Available\ food\ cropland, all\ uses + Available\ grazing\ land}{\sum_{i=1}^{N_g}\left(\frac{Required\ cultivated\ cropland\ _i}{Cropping\ intensity} + Required\ perennial\ cropland_i + Required\ grazing\ land_i\right) + Cropland\ used\ for\ grazing - Grazing\ land\ offset}$$

In each of the above formulas, N_g is the number of food subgroups for which there are data. The lowest of the three *Population fed* values is divided by the population size to estimate the proportion of the population that can be fed a given diet with current resource use efficiencies:

$$Carrying\ capapcity = \frac{Min(Population\ fed_{cultivated\ cropland}, Population\ fed_{all\ cropland}, Population\ fed_{all\ productive\ land})}{Population\ size}$$

The total annual quantity of land required to meet dietary demands (*Land use*) is solved by:

$$Land\ use = [(Required\ cultivated\ cropland\ /\ Cropping\ intensity) + Required\ forage\ land \times (1 + Cropland\ used\ for\ grazing)/Required\ forage\ land + Required\ grazing\ land \times (1 - Grazing\ land\ offset)/Required\ grazing\ land] \times Population\ size$$

The total quantity of each amendment (*b*: fertilizer nutrients, pesticides, and irrigation water) required to meet dietary demands (*Amendment use*) on an annual basis is solved by:

$$Amendment\ use_b = \sum_{i=1}^{N_f} Land\ use_i \times Amendment\ application\ rate_{bi},$$

where *Land use* is the quantity of land required for each food (*i*) on an annual per capita basis, *Amendment application rate* is quantity used per acre of each amendment (*b*) for each food on an annual basis, and N_f is the number of foods for which data are available.

Expanded output

The *Expanded output* worksheet is a new feature of the model that presents the amount of agricultural resources (land, fertilizer nutrients, pesticides, and irrigation water) associated with each of 208 foods included in the model. The embedded macro will export all of these data, along with the data from the *Output* worksheet, into a new spreadsheet that users can use to perform analyses in their statistical package of choice.

References

1. Peters C, Picardy J, Darrouzet-Nardi A, Wilkins J, Griffin T, Fick G. Carrying capacity of U.S. agricultural land: ten diet scenarios. Elementa 2016;4.
2. Kurtz JE, Woodbury PB, Ahmed ZU, Peters CJ. Mapping U.S. Food System Localization Potential: The Impact of Diet on Foodsheds. Environ Sci Technol 2020;54(19):12434-46.
3. US Department of Agriculture, Economic Research Service. *Loss-adjusted Food Availability (LAFA) data series.* 2023. Available at: https://www.ers.usda.gov/data-products/food-availability-per-capita-data-system/food-availability-per-capita-data-system/#Loss-Adjusted%20Food%20Availability (verified 06 November 2023).
4. US Department of Agriculture, Agricultural Research Service. *Food Patterns Equivalents Database (FPED).* 2023. Available at: https://www.ars.usda.gov/northeast-area/beltsville-md-bhnrc/beltsville-human-nutrition-research-center/food-surveys-research-group/docs/fped-methodology/ (verified 12 September 2024).
5. US Department of Agriculture, Agricultural Research Service. *Food and Nutrient Database for Dietary Studies.* 2001-2018. Available at: https://www.ars.usda.gov/northeast-area/beltsville-md-bhnrc/beltsville-human-nutrition-research-center/food-surveys-research-group/docs/fndds/ (verified 04 September 2020).
6. US Department of Health and Human Services and US Department of Agriculture. *Dietary Guidelines for Americans 2020-2025, Appendix 3.* Washington, DC2020. Available at: https://dietaryguidelines.gov/ (verified 20 January 2022).
7. US Department of Agriculture, Agricultural Research Service. *FoodData Central.* 2024. Available at: https://fdc.nal.usda.gov/ (verified 31 May 2021).
8. USDA Economic Research Service. *Weights, measures, and conversion factors for agricultural commodities and their products.* 1992. Available at website: http://www.ers.usda.gov/Publications/AH697/ (verified 25 April 2012).
9. US Department of Agriculture, Economic Research Service. *Oil Crops Yearbook.* Economics, Statistics, and Market Information System;2001-2010. Available at website: https://usda.library.cornell.edu/concern/publications/5x21tf41f?locale=en#release-items (verified 04 September 2023).

10. Peters CJ, Picardy JA, Darrouzet-Nardi A, Griffin TS. Feed conversions, ration compositions, and land use efficiencies of major livestock products in US agricultural systems. Agricultural Systems 2014;130(0):35-43.
11. National Research Council. *Nutrient requirements of animals collection.* 1994-2001. Available at: https://nap.nationalacademies.org/collection/63/nutrient-requirements-of-animals (verified 23 April 2023).
12. Dairy One Forage Lab. Interactive Feed Composition Libraries. Ithaca, New York, 2011.
13. Love DC, Asche F, Conrad Z, Young R, Harding J, Nussbaumer EM, Thorne-Lyman AL, Neff R. Food Sources and Expenditures for Seafood in the United States. Nutrients 2020;12(6).
14. Kroetz K, Luque GM, Gephart JA, Jardine SL, Lee P, Chicojay Moore K, Cole C, Steinkruger A, Donlan CJ. Consequences of seafood mislabeling for marine populations and fisheries management. PNAS 2020;117(48):30318-23.
15. MacLeod MJ, Hasan MR, Robb DHF, Mamun-Ur-Rashid M. Quantifying greenhouse gas emissions from global aquaculture. Sci Rep 2020;10(1):11679.
16. system Fafri. 2012-2022. Available at: https://www.feedipedia.org/ (verified 24 April 2023).
17. US Department of Agriculture, Economic Research Service. *Food Availability (LAFA) data series.* 2023. Available at: https://www.ers.usda.gov/data-products/food-availability-per-capita-data-system/food-availability-per-capita-data-system/#Loss-Adjusted%20Food%20Availability (verified 01 May 2023).
18. US Department of Agriculture, Foreign Agricultural Service. *Global Agricultural Trade System.* 2023. Available at: https://apps.fas.usda.gov/gats/ExpressQuery1.aspx (verified 01 May 2023).
19. US Department of Agriculture, National Agricultural Statistics Service. Quick Stats. 2023.
20. US Department of Agriculture NASS. *Table 1-77: Feed consumed by livestock and poultry, by type of feed, with quantity expressed in equivalent feeding value of corn, 2000-2009 in Agricultural Statistics.* Washington, DC2010.
21. US Department of Agriculture, Economic Research Service. *Major Land Uses: total grazing land, by region, State, and United States.* 2010. Available at: www.ers.usda.gov/data-products/major-land-uses.aspx (verified 01 May 2023).
22. US Department of Agriculture, Natural Resources Conservation Service. National Range and Pasture Handbook. . 2003.
23. National Research Council. *United States-Canadadian tables of feed composition.* 1982. Available at: http://www.nap.edu/openbook.php?record_id=1713&page=R1 (verified 03 May 2023).
24. US Department of Agriculture, National Agricultural Statistics Service. *Census of Agriculture.* Washington, DC2021. Available at: https://www.nass.usda.gov/AgCensus/ (verified 31 May 2021).
25. US Department of Agriculture, National Agricultural Statistics Service. *Census of Agriculture Methodology.* Washington, DC2021. Available at: https://www.nass.usda.gov/Publications/AgCensus/2017/index.php (verified 31 May 2021).

26. US Department of Agriculture, National Agricultural Statistics Service. *Agricultural Surveys.* 2019. Available at: http://www.nass.usda.gov/Quick_Stats/ (verified 31 May 2021).
27. US Department of Agriculture, National Agricultural Statistics Service. *About NASS Agricultural Surveys.* 2020. Available at: https://www.nass.usda.gov/Education_and_Outreach/Understanding_Statistics/index.php (verified 04 April 2020).
28. US Department of Agriculture, National Agricultural Statistics Service. *Farm and Ranch Irrigation Survey.* 2003-2013. Available at website: https://www.agcensus.usda.gov/Publications/Irrigation_Survey/ (verified 20 April 2020).
29. USDA E. Major Land Uses, Forest-use Land Tables. 1945-2007.
